# Methods for solving reasoning problems in abstract argumentation – A survey

**DOI:** 10.1016/j.artint.2014.11.008

**Published:** 2015-03

**Authors:** Günther Charwat, Wolfgang Dvořák, Sarah A. Gaggl, Johannes P. Wallner, Stefan Woltran

**Affiliations:** a Vienna University of Technology, Institute of Information Systems, Austria; b University of Vienna, Faculty of Computer Science, Austria; c Technische Universität Dresden, Institute of Artificial Intelligence, Germany

**Keywords:** Abstract argumentation, Algorithms, Argumentation systems

## Abstract

Within the last decade, abstract argumentation has emerged as a central field in Artificial Intelligence. Besides providing a core formalism for many advanced argumentation systems, abstract argumentation has also served to capture several non-monotonic logics and other AI related principles. Although the idea of abstract argumentation is appealingly simple, several reasoning problems in this formalism exhibit high computational complexity. This calls for advanced techniques when it comes to implementation issues, a challenge which has been recently faced from different angles. In this survey, we give an overview on different methods for solving reasoning problems in abstract argumentation and compare their particular features. Moreover, we highlight available state-of-the-art systems for abstract argumentation, which put these methods to practice.

## 1 Introduction

Argumentation is a highly interdisciplinary field with links to psychology, linguistics, philosophy, legal theory, and formal logic. Since the advent of the computer age, formal models of argument have been materialized in different systems that implement — or at least support — creation, evaluation, and judgment of arguments. However, until Dung's seminal paper on *abstract argumentation* [1], the heterogeneity of these approaches was severely hampering a strong and joint development of a field like “computational argumentation”. In fact, Dung's idea of evaluating arguments on an abstract level by taking only their inter-relationships into account, not only has been shown to underlie many of the earlier approaches for argumentation, but also uniformly captures several non-monotonic logics. Yet this second contribution located Argumentation as a sub-discipline of Artificial Intelligence [2]. The increasing significance of argumentation as a research area of its own has also been witnessed by the biennial COMMA Conference on Computational Models of Argument,^1^ which from the second meeting onwards provides sessions for software demonstrations of implemented systems, the IJCAI Workshop Series on Theory and Applications of Formal Argumentation (TAFA),^2^ the 2010 established Journal of Argument and Computation,^3^ or the Textbook on *Argumentation in Artificial Intelligence* [3].

One particular feature of abstract argumentation frameworks is their simple structure. In fact, abstract argumentation frameworks are just directed graphs where vertices play the role of arguments and edges indicate a certain conflict between the two connected arguments. These argumentation frameworks are usually derived during an *instantiation process* (see, e.g., [4,5]), where structured arguments are investigated with respect to their ability to contradict other such arguments; the actual notion of “contradicting” can be instantiated in many different forms (see, e.g., [6]). Having generated the framework in such a way, the process of “conflict-resolution”, i.e., the search for jointly acceptable sets of arguments, is then delegated to semantics which operate on the abstract level. Thus, semantics for argumentation frameworks have also been referred to as *calculi of opposition* [7].

One direction of research in abstract argumentation was devoted to develop the “right” forms of semantics (see, e.g., [8–10] where properties for argumentation semantics are proposed and evaluated). This has lead to what G. Simari has called a “*plethora of argumentation semantics*”.^4^ Today there seems to be agreement within the community that different semantics suit different applications, hence many of them are in use for a variety of application domains.^5^ It is clear that this situation implies that successful systems for abstract argumentation are expected to offer not only a single semantics.

The central role of abstract argumentation frameworks also boosted research on efficient procedures for this particular formalism. However, it was soon recognized that already these simple frameworks show high complexity (see, e.g., [12–14]); due to the link to non-monotonic logic and to logic programming in particular, this came without a huge surprise. Together with the fact that many different semantics exist, general implementation methods for abstract argumentation thus require

• a certain level of generality, such that not only a single semantics can be treated; and
• a sufficient level of efficiency to face the high inherent complexity of the problems at hand.

##### Scope of the survey

In this article, we present a selection of evaluation methods for abstract argumentation which we believe to meet these requirements. We group these methods into two categories: the *reduction approach* and the *direct approach*.

The underlying idea of the *reduction approach* is to exploit existing efficient software which has originally been developed for other purposes. To this end, one has to formalize the reasoning problems within other formalisms like constraint-satisfaction problems (CSP) [15], propositional logic [16] or answer-set programming (ASP) [17]. In this approach, the resulting argumentation systems directly benefit from the high level of sophistication today's systems for SAT (satisfiability in propositional logic) or ASP have reached. The reduction approach will be presented in detail in Section 3 of this article. Hereby, we will first focus on

• *SAT-based* argumentation systems. This direction has been advocated by Besnard and Doutre [18], and later extended by means of quantified propositional logic [19,20]. We will first discuss the theoretical underpinnings of this approach and then continue with an introduction to the CEGARTIX system [21] and the ArgSemSAT system [22], which both rely on iterative calls to SAT solvers for argumentation semantics of high complexity (i.e., being located on the second level of the polynomial hierarchy).
• *CSP-based* approach. Reductions to CSP have been addressed by Amgoud and Devred [23] and Bistarelli and Santini [24–28]; the latter work led to the development of the ConArg system. We introduce the formalization of argumentation frameworks in terms of CSPs, where the arguments of the given framework represent the variables of the CSP with domains of 0 and 1. The constraints then depend on the specific semantics.
• *ASP-based* approach. The use of this logic-programming paradigm to solve abstract argumentation problems has been initiated by several authors (the survey article by Toni and Sergot [29] provides a good overview). We focus here on the ASPARTIX approach [30] which in contrast to the aforementioned SAT methods relies on a query-based implementation where the argumentation framework to be evaluated is provided as an input database (from this point of view, the SAT or CSP methods can be seen as a compiler-like approach to abstract argumentation, while the ASP method acts like an interpreter). A large collection of such ASP queries is provided by the ASPARTIX system. We will discuss standard ways of ASP encodings, but also some recent methods which exploit advanced ASP techniques [31].

In the remainder of Section 3 we shall present the concepts behind other reduction-based approaches, for instance, the *equational approach* as introduced by Gabbay in [32] and the reductions to *monadic second order logic* as proposed in [33].

In Section 4, we collect methods and algorithms which have been developed from scratch (instead of using another formalism like SAT or ASP). The obvious disadvantage of this *direct approach* is due to the fact that existing technology cannot be directly employed. On the other hand, such argumentation-tailored algorithms ease the incorporation of short-cuts that are specific to the argumentation domain. In detail, we will discuss the following ideas:

• The *labeling approach* [34–38] gives a more fine-grained handle on the status of arguments when evaluated w.r.t. semantics and also provides a solid basis for dedicated algorithms. We present two different proposals for implementing the enumeration of preferred extensions, one along the lines of [35] and another following [34] using improvements from [36]. Furthermore, we discuss an algorithm dedicated to credulous reasoning with preferred semantics following the work of [38]. Labeling-based algorithms have been materialized in the ArguLab system as well as in Verheij's CompArg system.
• Characterizations via *Dialogue Games*. Here the acceptance status of an argument is given in terms of winning strategies in certain games on the argumentation framework. Typically such games are two-player games where one player, the proponent, argues in favor of the argument in question and a second player, the opponent, argues against it. Such games can be used to design algorithms [35,39], which are employed in systems like Dungine and Dung-O-Matic.
• Finally, we will take a look on *dynamic programming approaches* [40] which operate on decompositions of frameworks. Notably, the running times in this approach are not mainly dependent on the size of the given framework, but on a structural parameter. We focus on the parameter tree-width and the concept of tree decomposition. This method was first advocated by Dunne [41] and later realized in the dynPARTIX system [42].

As already hinted above, many of the methods we present have found their way into an available software system. Therefore, we will not only explain these methods in this survey, but shall also give the interested reader pointers to concrete systems which can be used to experiment. Section 5 contains a comparison of the systems w.r.t. their features (e.g. supported semantics and reasoning problems) and the underlying concepts. Some of the systems have been evaluated and compared w.r.t. their performance (see e.g., [31,36,43–45]), but no exhaustive performance comparisons have been done so far. In fact, an organized competition comparable to the ones from the areas of SAT [46] or ASP [47] is planned to take place in 2015 for the first time [48].^6^ Thus, we abstain here from a systematic comparison of the systems' performance.

To summarize, our goal is to introduce a selection of methods for evaluating abstract argumentation frameworks; we shall explain the key concepts in detail for selected semantics and give pointers to the literature for the remaining semantics or when it comes to more subtle aspects like optimization. Concerning abstract argumentation itself, we give a concise introduction in Section 2. For readers not familiar with abstract argumentation, we highly recommend the recent survey article by Baroni et al. [49].

Since the focus of this article is on the evaluation of semantics for Dung's abstract argumentation framework, advanced systems including instantiation (e.g., ASPIC [50] and Carneades [51]), assumption-based argumentation [52], or systems based on defeasible logic [53] are out of the scope of this article.^7^ Likewise, we will not consider the vast collection of extensions to Dung's frameworks,^8^ like value-based [56], bipolar [57], extended [58], constrained [59], temporal [60], practical [61], and fibring argumentation frameworks [62], as well as argumentation frameworks with recursive attacks [63], argumentation context systems [64], and abstract dialectical frameworks [65]. We also exclude abstract argumentation with uncertainty or weights here; recent articles by Hunter [66] and respectively Dunne et al. [67] introduce these variants in detail.

## 2 Background

In this section we introduce (abstract) argumentation frameworks [1] and recall the semantics we study in this paper (see also [10,49,68]).

Definition 1 An *argumentation framework (AF)* is a pair F=(A,R) where *A* is a set of arguments and R⊆A×A is the attack relation. The pair (a,b)∈R means that *a* attacks *b*. We say that an argument a∈A is *defended* (in *F*) by a set S⊆A if, for each b∈A such that (b,a)∈R, there exists a c∈S such that (c,b)∈R.

An argumentation framework can be represented as a directed graph.

**Fig. 1:**
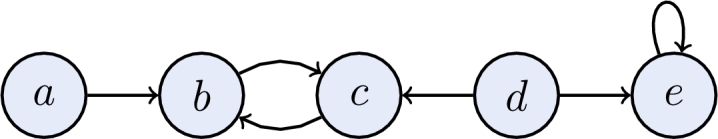
Example argumentation framework.

Example 1 Let F=(A,R) be an AF with A={a,b,c,d,e} and R={(a,b), (b,c), (c,b), (d,c), (d,e), (e,e)}. The corresponding graph representation is depicted in Fig. 1.

A semantics for argumentation frameworks is defined as a function *σ* which assigns to each AF F=(A,R) a set $\sigma (F)\subseteq 2^{A}$ of extensions.

We consider for *σ* the functions *naive*, *stb*, *adm*, *com*, *grd*, *prf*, *sem* and *stg* which stand for naive, stable, admissible, complete, grounded, preferred, semi-stable and stage extensions, respectively. Towards the definition of these semantics we introduce a few more formal concepts.

Definition 2 Given an AF F=(A,R), the *characteristic function* $F_{F}:2^{A}\to 2^{A}$ of *F* is defined as $F_{F}(S)=\{x\in A|x\text{ is defended by }S\}$. For a set S⊆A and an argument a∈A, we write $S{↣}^{R}a$ (resp. $a{↣}^{R}S$) in case there is an argument b∈S, such that (b,a)∈R (resp. (a,b)∈R). Furthermore, we write $S{↣̸}^{R}a$ (resp. $a{↣̸}^{R}S$) in case there is no argument b∈S, such that (b,a)∈R (resp. (a,b)∈R).

Moreover, for a set S⊆A, we denote the set of arguments attacked by (resp. attacking) *S* as $S_{R}^{⊕}=\{x{|S↣}^{R}x\}$ (resp. $S_{R}^{⊖}=\{x{|x↣}^{R}S\}$), and define the *range of S* as $S_{R}^{+}=S\cup S_{R}^{⊕}$ and the *negative range of S* as $S_{R}^{-}=S\cup S_{R}^{⊖}$.

The next definition formally defines the semantics we will focus on in this survey. All of them are based on conflict-free sets, i.e. it is not allowed to jointly accept arguments which are adjacent in the framework. Different additional criteria are then used for the concrete definition: naive extensions are just maximal (with respect to set-inclusion) conflict-free sets, stable extensions have to attack all other arguments, admissible sets defend themselves from attackers, complete extensions in addition have to contain all defended arguments. The grounded extension is given by the subset-minimal complete extension. Preferred extensions are subset-maximal admissible sets (equivalently: subset-maximal complete extensions); finally, semi-stable and stage extensions are characterized by maximizing the concept of range.

Definition 3 Let F=(A,R) be an AF. A set S⊆A is *conflict-free* (*in F*), if there are no a,b∈S, such that (a,b)∈R. cf(F) denotes the collection of conflict-free sets of *F*. For a conflict-free set S∈cf(F), it holds that

• S∈naive(F), if there is no T∈cf(F) with T⊃S;
• S∈stb(F), if $S_{R}^{+}=A$;
• S∈adm(F), if $S\subseteq F_{F}(S)$;
• S∈com(F), if $S=F_{F}(S)$;
• S∈grd(F), if S∈com(F) and there is no T∈com(F) with T⊂S;
• S∈prf(F), if S∈adm(F) and there is no T∈adm(F) with S⊂T;
• S∈sem(F), if S∈adm(F) and there is no T∈adm(F) with $S_{R}^{+}\subset T_{R}^{+}$;
• S∈stg(F), if there is no T∈cf(F), with $S_{R}^{+}\subset T_{R}^{+}$.

**Fig. 2:**
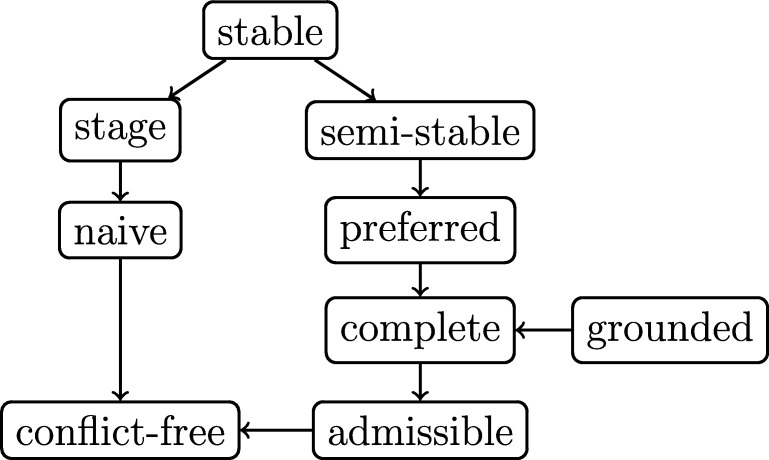
Relations between argumentation semantics: An arrow from a semantics *σ* to another semantics *τ* denotes that each *σ*-extension is also a *τ*-extension.

We recall that for each AF *F*, the grounded semantics yields a unique extension, the grounded extension, which is the least fixed-point of the characteristic function $F_{F}$. Furthermore, Fig. 2 shows the relations between the aforementioned semantics. The figure is complete in the sense that if there is no arrow from semantics *σ* to semantics *τ*, then there is some AF *F* such that σ(F)⊈τ(F). For all semantics *σ* we introduced here, except stable semantics, it holds that for any AF *F* we have σ(F)≠∅.

Example 2 Consider the AF from Example 1. Then: cf(F)={∅,{a},{b},{c},{d},{a,c},{a,d},{b,d}}; naive(F)={{a,c},{a,d},{b,d}}; adm(F)={∅,{a},{d},{a,d}}; and stb(F)=com(F)=grd(F)=prf(F)=sem(F)=stg(F)={{a,d}}.

##### Labeling-based semantics

So far we have considered so-called extension-based semantics. However, there are several approaches defining argumentation semantics via certain kinds of labelings instead of extensions. As an example we consider the popular approach by Caminada and Gabbay [69] and in particular their complete labelings. Basically, such a labeling is a three-valued function that assigns one of the labels *in*, *out* and *undec* to each argument, with the intuition behind these labels being the following. An argument is labeled with: *in* if it is accepted, i.e., it is defended by the *in* labeled arguments; *out* if there are strong reasons to reject it, i.e., it is attacked by an accepted argument; *undec* if the argument is undecided, i.e., neither accepted nor attacked by accepted arguments. We denote labeling functions L also by triples $\left(L_{\mathrm{in}},L_{\mathrm{out}},L_{\text{undec}}\right)$, where $L_{\mathrm{in}}$ is the set of arguments labeled by *in*, $L_{\mathrm{out}}$ is the set of arguments labeled by *out* and $L_{\text{undec}}$ is the set of arguments labeled by *undec*.

As an example, we give the definition of complete labelings from [69].

Definition 4 Given an AF F=(A,R), a function L:A→{in,out,undec} is a *complete labeling* iff the following conditions hold:

• L(a)=in iff for each *b* with (b,a)∈R, L(b)=out.
• L(a)=out iff there exists *b* with (b,a)∈R, L(b)=in.

There is a one-to-one mapping between complete extensions and complete labelings, such that the set of arguments labeled with *in* corresponds to the complete extension and the arguments labeled with *out* correspond to the arguments attacked by the complete extension. Having complete labelings at hand we can also characterize preferred labelings as follows:

Definition 5 Given an AF F=(A,R). The *preferred labelings* are those complete labelings where $L_{\mathrm{in}}$ is ⊆-maximal among all complete labelings.

Right by the definitions, we have the same one-to-one mapping between preferred extensions and preferred labelings as for complete semantics. One can define labeling-based versions for all of our semantics (see [69]), but this is out of the scope of this survey.

##### Reasoning in argumentation frameworks

We recall the most important reasoning problems for AFs: Given an argumentation framework *F* and a semantics *σ*, ${\mathrm{Enum}}_{\sigma }(F)$ results in an enumeration of all extensions. A simpler notion is ${\mathrm{Count}}_{\sigma }(F)$, which only counts the number of extensions. Query-based problems are ${\mathrm{Cred}}_{\sigma }(a,F)$ and ${\mathrm{Skept}}_{\sigma }(a,F)$ for deciding credulous (respectively skeptical) acceptance of an argument *a*. The former returns *yes* if *a* is contained in at least one extension under *σ*, while for the latter to return *yes*, *a* must be contained in all extensions under *σ*. Finally, we also consider the problem ${\mathrm{Ver}}_{\sigma }(S,F)$ of verifying a given extension, i.e., testing whether a given set *S* is a *σ*-extension of *F*. This problem typically occurs as a subroutine of a reasoning procedure.

Definition 6 Given an AF F=(A,R), a semantics *σ* and an argument a∈A, then

• ${\mathrm{Enum}}_{\sigma }(F)=\sigma (F)$
• ${\mathrm{Count}}_{\sigma }(F)=|\sigma (F)|$
• ${\mathrm{Cred}}_{\sigma }(a,F)=\left\{ \begin{array}{cc} \text{yes} & \text{if }a\in {⋃}_{S\in \sigma (F)}S\\ \text{no} & \text{otherwise} \end{array}\right.$
• ${\mathrm{Skept}}_{\sigma }(a,F)=\left\{ \begin{array}{cc} \text{yes} & \text{if }a\in {⋂}_{S\in \sigma (F)}S\\ \text{no} & \text{otherwise} \end{array}\right.$
• $Ver_{\sigma }(S,F)=\left\{ \begin{array}{cc} \text{yes} & \text{if }S\in \sigma (F)\\ \text{no} & \text{otherwise} \end{array}\right.$

Example 3 Consider the AF *F* given in Example 1. For naive semantics, the reasoning problems result in ${\mathrm{Enum}}_{\mathrm{naive}}(F)=\{\{a,c\},\{a,d\},\{b,d\}\}$ and ${\mathrm{Count}}_{\mathrm{naive}}(F)=3$. Furthermore, for argument *a* we obtain ${\mathrm{Cred}}_{\mathrm{naive}}(a,F)=\text{yes}$ and ${\mathrm{Skept}}_{\mathrm{naive}}(a,F)=\text{no}$. For preferred semantics, *F* has a single extension ${\mathrm{Enum}}_{\mathrm{prf}}(F)=\{\{a,d\}\}$, ${\mathrm{Count}}_{\mathrm{prf}}(F)=1$, and thus credulous and skeptical acceptance coincide (e.g., ${\mathrm{Cred}}_{\mathrm{prf}}(a,F)={\mathrm{Skept}}_{\mathrm{prf}}(a,F)=\text{yes}$).

Next, let us turn to the complexity of reasoning in abstract argumentation frameworks. We assume the reader has knowledge about standard complexity classes, i.e., P, NP and L (logarithmic space). Furthermore, we briefly recapitulate the concept of oracle machines and related complexity classes. Let C denote some complexity class. By a C-oracle machine we mean a (polynomial time) Turing machine which can access an oracle that decides a given (sub)-problem in C within one step. We denote such machines as ${\mathrm{NP}}^{C}$ if the underlying Turing machine is non-deterministic. The class $\Sigma_{2}^{P}={\mathrm{NP}}^{\mathrm{NP}}$ thus denotes the set of problems which can be decided by a non-deterministic polynomial time algorithm that has (unrestricted) access to an NP-oracle. The class $\Pi_{2}^{P}={\mathrm{coNP}}^{\mathrm{NP}}$ is defined as the complementary class of $\Sigma_{2}^{P}$, i.e., $\Pi_{2}^{P}=\mathrm{co}\Sigma_{2}^{P}$. The relation between the complexity classes is as follows:

$$L\subseteq P\subseteq \begin{array}{c} \mathrm{NP}\\ \mathrm{coNP} \end{array}\subseteq \begin{array}{c} \Sigma_{2}^{P}\\ \Pi_{2}^{P} \end{array}$$

The computational complexity of credulous and skeptical reasoning has been studied extensively in the literature (see [70] for a starting point). Table 1 summarizes the computational complexity classifications of the defined decision problems [68,12,1,13,71,14,72,73], where C-c denotes that the corresponding problem is complete for class C.

## 3 Reduction-based approaches

In this section we will discuss reduction-based approaches in abstract argumentation. As implied by the name, these methods reduce or translate a reasoning problem to another, typically to another formalism. From a computational point of view, we assure that this reduction is efficiently computable, i.e., achievable in polynomial time, and that the answer for the original problem instance can be immediately obtained from the answer to the new problem instance. Such methods offer the great benefit of exploiting existing and highly sophisticated solvers for well-known and well-studied problem domains.

Naturally, reduction-based methods can be distinguished by the target system. Many such approaches have been studied for abstract argumentation ranging from propositional logic [19,18,21,20], constraint satisfaction problems (CSP) [27,23,26,28] and answer-set programming (ASP) [30,74–76] to equational systems [32,77]. We will give an overview of these approaches and in particular focus on the first three very prominent target systems, the reductions to propositional logic, CSP and ASP.

### 3.1 Propositional-logic based approach

Propositional logic is the prototypical target system for many approaches based on reductions, as the Boolean SAT problem is well studied and moreover accompanied with many mature and efficient solvers such as MiniSat [78] and GRASP [79].

First, we recall the necessary background of Boolean logic and quantified Boolean formulae (QBF) since they serve as our target systems.

The basis of propositional logic is a set of propositional variables P, to which we also refer to as atoms. Propositional formulae are built as usual from the connectives ∧,∨,→ and ¬, denoting the logical conjunction, disjunction, (material) implication and negation respectively. We use the truth constants ⊤ to denote *true* and ⊥ for *false*. In addition, we consider quantified Boolean formulae with the universal quantifier ∀ and the existential quantifier ∃ (both over atoms), that is, given a formula *ϕ*, then *Qpϕ* is a QBF, with Q∈{∀,∃} and p∈P. Furthermore, $Q\{p_{1},\ldots ,p_{n}\}\phi$ is a shorthand for $Qp_{1}\cdots Qp_{n}\phi$. The order of variables in consecutive quantifiers of the same type does not matter.

A propositional variable *p* in a QBF *ϕ* is free if it does not occur within the scope of a quantifier *Qp* and bound otherwise. If *ϕ* contains no free variable, then *ϕ* is said to be closed and otherwise open. We will write ϕ[p/ψ] to denote the result of uniformly substituting each free occurrence of *p* with *ψ* in formula *ϕ*.

An interpretation I⊆P defines for each propositional variable a truth assignment where p∈I indicates that *p* evaluates to true while p∉I indicates that *p* evaluates to false. This generalizes to arbitrary formulae in the standard way: Given a formula *ϕ* and an interpretation *I*, then *ϕ* evaluates to true under *I* (i.e., *I* satisfies *ϕ*) if one of the following holds (with p∈P).

• ϕ=p and p∈I
• ϕ=¬p and p∉I
• $\phi =\psi_{1}\wedge \psi_{2}$ and both $\psi_{1}$ and $\psi_{2}$ evaluate to true under *I*
• $\phi =\psi_{1}\vee \psi_{2}$ and one of $\psi_{1}$ and $\psi_{2}$ evaluates to true under *I*
• $\phi =\psi_{1}\to \psi_{2}$ and $\psi_{1}$ evaluates to false or $\psi_{2}$ evaluates to true under *I*
• ϕ=∃pψ and one of ψ[p/⊤] and ψ[p/⊥] evaluates to true under *I*
• ϕ=∀pψ and both ψ[p/⊤] and ψ[p/⊥] evaluate to true under *I*.

If an interpretation *I* satisfies a formula *ϕ*, denoted by I⊨ϕ, we say that *I* is a model of *ϕ*.

The approaches in Section 3.1.1 and Section 3.1.2 share the basic idea of translating a given AF, a semantics and a reasoning problem to a propositional formula, thereby reducing the problem to Boolean logic. In general this works by either inspecting the models of the resulting formula, which are in correspondence to the extensions of the AF, or deciding whether a formula is satisfiable or unsatisfiable, to solve query-based reasoning. Note that we restrict ourselves here to the semantics which we consider to be sufficient for illustrating the main concepts. In general, the approaches can be applied to many other semantics.

#### 3.1.1 Reductions to propositional logic

The first reduction-based approach [18,20] we consider here uses propositional logic formulae (without quantifiers) to encode the problem of finding admissible sets. Given an AF F=(A,R), for each argument a∈A a propositional variable $v_{a}$ is used. Then, S⊆A is an extension under semantics *σ* iff $\{v_{a}|a\in S\}⊨\phi$, with *ϕ* being a propositional formula that evaluates AF *F* under semantics *σ* (below we will present in detail how to translate AFs into formulae). Formally, the correspondence between sets of extensions and models of a propositional formula can be defined as follows.

Definition 7 Let $S\subseteq 2^{A}$ be a collection of sets of arguments and let $I\subseteq 2^{P}$ be a collection of interpretations. We say that S and I correspond to each other, in symbols S≅I, if

1. for each S∈S, there exists an I∈I, such that $\{a|v_{a}\in I,a\in A\}=S$;
2. for each I∈I, there exists an S∈S, such that $\{a|v_{a}\in I,a\in A\}=S$; and
3. |S|=|I|.

Given an AF F=(A,R) the following formula can be used to solve the enumeration problem of admissible semantics.

(1) $${\mathrm{adm}}_{A,R}=\underset{a\in A}{⋀}\left(\left(v_{a}\to \underset{(b,a)\in R}{⋀}\neg v_{b}\right)\wedge \left(v_{a}\to \underset{(b,a)\in R}{⋀}\left(\underset{(c,b)\in R}{⋁}v_{c}\right)\right)\right)$$

The models of ${\mathrm{adm}}_{A,R}$ now correspond to the admissible sets of *F*, i.e., ${\mathrm{Enum}}_{\mathrm{adm}}(F)≅\{M|M⊨{\mathrm{adm}}_{A,R}\}$. Taken into consideration that by definition a satisfiable formula has infinitely many models (thus violating item three in Definition 7), it is now possible to restrict the set of models to those containing only atoms occurring in the formula. The first conjunction in (1) ensures that the resulting set of arguments is conflict-free, that is, whenever we accept an argument *a* (i.e., $v_{a}$ evaluates to true under a model), all its attackers cannot be selected any further. The second conjunct expresses the defense of arguments by stating that, if we accept *a*, then for each attacker *b*, some defender *c* must be accepted as well. Note that an empty conjunction is treated as ⊤, whereas the empty disjunction is treated as ⊥.

Example 4 The propositional formula for admissible sets of the framework F=(A,R) in Example 1 is given by

(2) $${\mathrm{adm}}_{A,R}\equiv (v_{a}\to ⊤)\wedge$$

(3) admA,R≡(vb→(¬va∧¬vc))∧

(4) admA,R≡(vc→(¬vb∧¬vd))∧

(5) admA,R≡(vd→⊤)∧

(6) admA,R≡(ve→(¬vd∧¬ve))∧

(7) admA,R≡(va→⊤)∧

(8) admA,R≡(vb→(⊥∧(vb∨vd)))∧

(9) admA,R≡(vc→((va∨vc)∧⊥))∧

(10) admA,R≡(vd→⊤)∧

(11) admA,R≡(ve→(⊥∧vd))

Lines (2) to (6) encode the conflict-free property, while lines (7) to (11) ensure that arguments in an admissible set are defended. Note that for convenience, the conjuncts are arranged in a different order than in the definition of ${\mathrm{adm}}_{A,R}$. Consider for instance argument *b*. Line (3) specifies that if we accept *b* we cannot accept *a* and *c* anymore (conflict-free property). Likewise, line (8) states that *b* can only be accepted in case it is defended against its attackers. For the attacker *c* either *b* itself or *d* must be accepted. However, since attacker *a* is not attacked by any other argument, there is no model of ${\mathrm{adm}}_{A,R}$ where $v_{b}$ evaluates to true.

Another interesting translation to capture semantics of AFs within propositional logic is done by Gabbay [80]. Here, a correspondence between AFs and propositional logic is shown via the Peirce–Quine dagger (“nor”) connective.

Furthermore, several papers deal with the converse translation, i.e. translating a Boolean formula in CNF to an AF. Similar as before, for each atom in a formula a corresponding argument is constructed. Accepting such an argument, e.g. under stable semantics, is then interpreted as setting the atom to true. The result is a correspondence between extensions under a specific semantics and satisfying assignments of the formula. Usually, these translations incorporate auxiliary arguments, which are used to simulate the logical connectives. In [12,13] and [81] such methods are studied and used to show complexity bounds or for translations between formalisms.

#### 3.1.2 Reductions to quantified Boolean formulae

Problems beyond NP require a more expressive formalism than Boolean logic. Preferred semantics, for example, is defined as subset-maximal admissible (or complete) sets. Intuitively, we can compute a preferred extension by searching for an admissible set and additionally making sure that there is no proper superset which is also admissible. In order to express subset maximality directly inside the logic, a universal (or, equivalently, a negated existential) quantifier can be used, making quantified Boolean formulae a well-suited formalism. It is possible to specify preferred semantics in QBFs either via an extension-based, or a labeling-based approach.

First, we consider the extension-based approach from [20]. Here, we encode the maximality check with an auxiliary formula. For convenience we denote by $A^{\prime }=\{a^{\prime }|a\in A\}$ the set of renamed arguments in *A*. Likewise, we define a renaming for the attack relation as $R^{\prime }=\{(a^{\prime },b^{\prime })|(a,b)\in R\}$. The following defines a shorthand for comparing two sets of atoms an interpretation is defined upon with respect to the subset-relation.

$$A<A^{\prime }=\underset{a\in A}{⋀}(v_{a}\to v_{a^{\prime }})\wedge \neg \underset{a^{\prime }\in A^{\prime }}{⋀}(v_{a^{\prime }}\to v_{a})$$

In other words, this formula ensures that any model $M⊨(A<A^{\prime })$ satisfies $\left\{a\in A|v_{a}\in M\}\subset \{a\in A|v_{a^{\prime }}\in M\right\}$. Now we can state the QBF for preferred extensions. Let the quantified variables be $A_{v}^{\prime }=\{v_{a^{\prime }}|a^{\prime }\in A^{\prime }\}$.

(12) $${\mathrm{prf}}_{A,R}={\mathrm{adm}}_{A,R}\wedge \neg \exists A_{v}^{\prime }\left(\left(A<A^{\prime }\right)\wedge {\mathrm{adm}}_{A^{\prime },R^{\prime }}\right)$$

In short, we check whether the accepted arguments form an admissible set and whether there exists a proper superset of it which is also admissible. If the former check succeeds and in the latter no such set exists, then we have found a preferred extension. For an arbitrary AF F=(A,R), its preferred extensions are in a 1-to-1 correspondence to the models of ${\mathrm{prf}}_{A,R}$, i.e., ${\mathrm{Enum}}_{\mathrm{prf}}(F)≅\{M|M⊨{\mathrm{prf}}_{A,R}\}$.

The second approach is based on complete labelings (see Definition 4) instead of extensions [19].^9^ To this end, we employ four-valued interpretations to express more than two possible states for each argument. In addition to the truth values true and false we also add values undecided and inconsistent. The three labelings in, out and undecided correspond to the first three truth values. The whole approach can be encoded in classical two-valued QBFs. Hereby, the truth value of p∈P is encoded via $p^{⊕}$ and $p^{⊖}$. Now every classical two-valued interpretation assigns values to these two atoms as usual. For two variables we have four different cases, which correspond to the four truth values: $\{p^{⊕},p^{⊖}\}\subseteq I$ is interpreted as assigning inconsistent to *p*, true (resp. false) is assigned to *p* if only $p^{⊕}$ (resp. $p^{⊖}$) is in *I*, and undecided is assigned if neither $p^{⊕}$ nor $p^{⊖}$ is in *I*.

For preferred semantics the encoding is more complex than (12), but the ideas are similar. We begin with formulae for the four truth values. Note that we slightly adapted the representation and formulae from [19] to better match the previous encodings, but the important concepts remain the same.

$$\mathrm{val}(p,v)=\left\{ \begin{array}{cc} p^{⊕}\wedge p^{⊖} & \text{if}\;v=i\\ p^{⊕}\wedge \neg p^{⊖} & \text{if}\;v=t\\ \neg p^{⊕}\wedge p^{⊖} & \text{if}\;v=f\\ \neg p^{⊕}\wedge \neg p^{⊖} & \text{if}\;v=u \end{array}\right.$$

Here, val(p,v) encodes the four possible truth values for a virtual atom *p* that can be referred to on a sort of meta-level. Actually, instead of *p* the auxiliary atoms $p^{⊕}$ and $p^{⊖}$ are present in the concrete formula. Using this concept, we can specify the labeling formula for each argument in an AF F=(A,R).

(13) $${\mathrm{lab}}_{A,R}^{t}(a)=\mathrm{val}(v_{a},t)\to \underset{(b,a)\in R}{⋀}\mathrm{val}(v_{b},f)$$

(14) $${\mathrm{lab}}_{A,R}^{f}(a)=\mathrm{val}(v_{a},f)\to \underset{(b,a)\in R}{⋁}\mathrm{val}(v_{b},t)$$

(15) $${\mathrm{lab}}_{A,R}^{u}(a)=\mathrm{val}(v_{a},u)\to \left(\left(\neg \underset{(b,a)\in R}{⋀}\mathrm{val}(v_{b},f)\right)\;\wedge \left(\neg \underset{(b,a)\in R}{⋁}\mathrm{val}(v_{b},t)\right)\right)$$

These formulae reflect Definition 4: The formulae (13), (14) and (15) encode the in, out and undecided labelings, respectively. For example, (13) can be interpreted in the following way: If an argument *a* is set to true, then all its attackers must be false. (14) can be interpreted similarly, except that if an atom denotes that an argument is false, then one of its attackers must be true. Finally, (15) states that for any argument to which we assign undecided, it cannot be the case that all its attackers are false or that one of them is true.

Three values are sufficient to reflect the three labelings. To avoid problems with the fourth truth value (inconsistent), we exclude it from occurring in the evaluation by the following formula.

$$3{\mathrm{val}}_{A}=\underset{a\in A}{⋀}\neg \mathrm{val}(v_{a},i)$$

Now, complete extensions are characterized by the following formula. We will use *L* as superscript in ${\mathrm{com}}_{A,R}^{L}$ to denote that this formula handles labelings instead of extensions.

$${\mathrm{com}}_{A,R}^{L}:=3{\mathrm{val}}_{A}\wedge \underset{a\in A}{⋀}\left({\mathrm{lab}}_{A,R}^{t}(a)\wedge {\mathrm{lab}}_{A,R}^{f}(a)\wedge {\mathrm{lab}}_{A,R}^{u}(a)\right)$$

The formula ${\mathrm{com}}_{A,R}^{L}$ expresses that all the arguments are assigned either true, false or undecided via the $3{\mathrm{val}}_{A}$ sub-formula. For each argument, the three conjuncts on the right of the formula encode implications which ensure that the labels are assigned as specified for complete labelings. For example, if *a* is true, then all its attackers must be false. By applying this, one can encode complete labelings and hence complete extensions. Preferred extensions (or labelings) are expressed as before by subset maximization.

(16) A<A′L=⋀a∈A(val(va,t)→val(va′,t))∧¬⋀a′∈A′(val(va′,t)→val(va,t))

Then, similar as in ${\mathrm{prf}}_{A,R}$, the preferred extensions or their labelings can be encoded with a QBF as follows, with the quantified atoms $A_{v}^{\prime }=\{v_{a^{\prime }}^{⊕},v_{a^{\prime }}^{⊖}|a^{\prime }\in A^{\prime }\}$.

(17) prfA,RL=comA,RL∧¬∃Av′((A<A′L)∧comA′,R′L)

For an AF F=(A,R) the following notion of correspondence holds: Let the set of atoms evaluated to true under the four-valued interpretation be $M^{t}=\{p|p^{⊕}\in M,p^{⊖}\notin M\}$, then ${\mathrm{Enum}}_{\mathrm{prf}}(F)≅\{M^{t}|M⊨{\mathrm{prf}}_{A,R}^{L}\}$. Note that ${\mathrm{prf}}_{A,R}^{L}$ differs from ${\mathrm{prf}}_{A,R}$ not only by using a labeling-based approach, but also by maximizing complete labelings rather than admissible sets.

Utilizing the expressive power of quantifiers and the labeling approach, the authors of [19] also encode a range of other semantics, for instance semi-stable reasoning, where one can apply the same idea as outlined above, but instead of maximizing the arguments that are in, the arguments that are labeled undecided are minimized.

This results in a general system for encoding many semantics, but one has to be careful with choosing the right target system. For example, grounded semantics can easily be specified in this formalism using a QBF, but computing the grounded extension can be done using an algorithm with polynomial running time. Thus, an appropriate encoding would yield a QBF from a fragment which is known to be efficiently decidable, for instance, 2-QBF (the generalization of Krom formulae to QBFs). However, we are not aware of any work which deals with such “complexity-sensitive” encodings in terms of QBFs.

#### 3.1.3 Iterative application of SAT solvers

The last propositional-logic based approach we outline here is based on the idea of iteratively searching for models of propositional formulae and has been instantiated in the systems ArgSemSAT [82,22] and CEGARTIX [21,83]. The idea is to use an algorithm which iteratively constructs formulae and searches for models of these formulae. A new formula is generated based on the model of the previous one (or based on the fact that the previous formula is unsatisfiable). At some point the algorithm reaches a final decision and terminates. This is in contrast to so-called monolithic encodings, which formulate the whole problem in a single formula. The encodings in previous sections are examples for such monolithic encodings. The iterative approach is suitable when the problem to be solved cannot be decided in general (under standard complexity theoretic assumptions) by the satisfiability of a single propositional formula (constructible in polynomial time) without quantifiers. This is, for instance, the case with skeptical acceptance under preferred semantics where the corresponding decision problem is $\Pi_{2}^{P}$ complete. Instead of reducing the problem to a single QBF formula, we delegate the solving task in the iterative scheme to an algorithm querying a SAT solver multiple times.

The algorithms for preferred semantics work roughly as follows. To compute preferred extensions we traverse the search space of a computationally simpler semantics. For instance, we can iteratively search for admissible sets or complete extensions and iteratively extend them until we reach a maximal set, which is a preferred extension. By generating a new candidate admissible set/complete extension, which is not contained in an already visited preferred extension we can enumerate all preferred extensions in this manner. This allows answering credulous and skeptical reasoning as well.

For deciding e.g. skeptical acceptance of an argument under preferred semantics one requires in general an exponential number of calls to the SAT solver (under standard complexity theoretic assumptions). However, the number of SAT calls in the iterative SAT scheme is dependent on the number of preferred extensions of the given AF, see [21].

In the following, we first sketch the CEGARTIX approach from [21] for skeptical acceptance under preferred semantics and subsequently outline the PrefSat approach [82], implemented in the ArgSemSAT system, for enumerating all preferred extensions. Again, we slightly adapted the algorithms for a uniform setting and presentation.

Algorithm 1 decides skeptical acceptance under preferred semantics of an argument *a* in an AF *F*. The idea is to proceed from one preferred extension to the next and checking whether *a* is in one of the extensions. This is encoded in the outer while loop, lines 2 to 11. The models of formula *ϕ* represent the remaining admissible sets in the current state of the algorithm. In the beginning, *ϕ* encodes all admissible sets of *F*. We start with an admissible set and iteratively extend it while making sure that *a* is not accepted in this admissible set. This is done in the second loop (lines 3 to 5) and by adding $\neg v_{a}$ to the query. The formula $\psi^{I}$ incorporates the model *I* and states that a model of it must still correspond to an admissible set, but also has to be a superset of the current one, specified by *I*.

If we cannot add further arguments to the admissible set, we check whether we can extend it with having *a* inside, in line 6. If this is the case, every preferred extension that is a superset of the current admissible set contains *a*. Hence, we can proceed to a different admissible set not containing *a*. In case we cannot add *a* to the admissible set, we have found a preferred extension without *a*, hereby refuting its skeptical acceptance in *F*. In the former case (*I* does not represent a preferred extension) we strengthen the main query *ϕ* by adding $\gamma^{I}$ in line 7, stating that at least one argument currently not accepted in *I* must be accepted from now on. This ensures that in future iterations we compute admissible sets that are not contained in previously found preferred extensions.

The formulae are defined as follows.

$$\psi^{I}(A,R)={\mathrm{adm}}_{A,R}\wedge \underset{a\in A,v_{a}\in I}{⋀}v_{a}\wedge \left(\underset{a\in A,v_{a}\notin I}{⋁}v_{a}\right)$$

$$\gamma^{I}=\underset{a\in A,v_{a}\notin I}{⋁}v_{a}.$$

Example 5 For the AF *F* from Example 1 we can check the skeptical acceptance of *b*. The condition of the first loop is satisfied as there exist the admissible sets ∅, {a}, {d} and {a,d} in *F*. The algorithm now non-deterministically selects one of the admissible sets. Say we pick ∅. The second while loop then creates a subset maximal admissible set (excluding *b*) in two iterations, say first adding *a* and then *d*. As {a,d} is now subset maximal, the second loop terminates. Since this set cannot be extended if we allow to also accept *b*, we know that we have found a preferred extension. This means we refute the skeptical acceptance of *b*.

The PrefSat approach [82] is designed to enumerate all preferred extensions utilizing a similar idea. Hereby, also a simpler semantics for traversing the search space is used, but the encodings rely on the concept of labellings (see also Section 4.1). We outline the PrefSat procedure in Algorithm 2.

PrefSat encodes labelings of an AF F=(A,R) by generating three variables per argument, i.e., the set of variables in the constructed formula are $\left\{I_{a},O_{a},U_{a}|a\in A\right\}$. In the final result these variables correspond naturally to a labeling. In particular, a three-valued labeling *K* corresponds to a model *J* if K=(I,O,U) with $I=\{a|I_{a}\in J\}$, $O=\{a|O_{a}\in J\}$ and $U=\{a|U_{a}\in J\}$. The following constraint encodes that for every argument exactly one labeling is assigned.

$$\underset{a\in A}{⋀}\left((I_{a}\vee O_{a}\vee U_{a})\wedge (\neg I_{a}\vee \neg O_{a})\wedge (\neg I_{a}\vee \neg U_{a})\wedge (\neg O_{a}\vee \neg U_{a})\right)$$

Furthermore, one can encode the conditions for a labeling to be complete by conjoining certain subformulae. For instance, the formula

$$\underset{x\in A,\exists (y,x)\in R}{⋀}\left(\underset{(x^{\prime },x)\in R}{⋀}(\neg I_{x}\vee O_{x^{\prime }})\right)$$

encodes that we can accept *x* if each attacker is out. The remaining constraints for a labeling to be complete are encoded similarly. Several equivalent formulae for encoding complete labelings have been investigated by Cerutti et al. [82]. Let ${\mathrm{com}}_{A,R}^{\prime }$ be one of these choices for encoding complete labelings. The formulae for Algorithm 2 are then defined as follows.

$$\psi^{J}(A,R)={\mathrm{com}}_{A,R}^{\prime }\wedge \underset{a\in A,I_{a}\in J}{⋀}I_{a}\wedge \left(\underset{a\in A,I_{a}\notin J}{⋁}I_{a}\right)$$

$$\gamma^{J}=\underset{a\in A,I_{a}\notin J}{⋁}I_{a}.$$

Algorithm 2 traverses the space of complete labelings in two while loops. The outer while loop computes candidate complete labelings, which are not smaller than previously found preferred labelings (when comparing only the arguments assigned *in*). The inner while loop searches for a maximal preferred labeling. If the inner while loop terminates, then the corresponding preferred labeling/extension is added to the solution set S. In line 8 we exclude smaller complete labelings from subsequent iterations. In [82] the algorithm for enumerating all preferred extensions contains further refinements, such as restricting the search space to non-empty complete extensions.

#### 3.1.4 Reasoning problems

The first two reductions presented in Section 3.1.1 and Section 3.1.2 immediately solve problems of enumerating extensions. Deciding credulous and skeptical reasoning is typically easy to achieve. In order to decide ${\mathrm{Cred}}_{\sigma }(a,F)$ one can conjoin the base formula with an atom corresponding to the acceptance of argument *a*. If there exists a model, *a* is credulously accepted. Adding the atom for *a* in negated form to the formula decides if *a* is not skeptically accepted, i.e., if there exists a model, an extension does not contain *a*.

Similarly, the iterative SAT scheme of Algorithm 1 (see Section 3.1.3) can be adapted to solve credulous reasoning by adding the atom to be queried positively instead of negatively in line 3. Regarding the enumeration problem, Algorithm 2 enumerates all preferred extensions using iterative SAT.

Counting the number of extensions cannot be easily encoded in the formulae, but the SAT solver itself may offer this feature by counting the number of models.

### 3.2 CSP-based approach

In this subsection we consider reductions to Constraint Satisfaction Problems (CSPs) [84], which allow solving combinatorial search problems. The approach of CSP is inherently related to propositional logic reductions as introduced in Subsection 3.1.1, see also [85] for a formal analysis of the relation between the two approaches.

A CSP can generally be described by a triple (X,D,C), where $X=\{x_{1},\ldots ,x_{n}\}$ is the set of variables, $D=\{D_{1},\ldots ,D_{n}\}$ is a set of finite domains for the variables and $C=\{c_{1},\ldots ,c_{m}\}$ a set of constraints. Each constraint $c_{i}$ is a pair $\left(h_{i},H_{i}\right)$ where $h_{i}=(x_{i1},\ldots ,x_{ik})$ is a *k*-tuple of variables and $H_{i}$ is a *k*-ary relation over *D*. In particular, $H_{i}$ is a subset of all possible variable values representing the allowed combinations of simultaneous values for the variables in $h_{i}$. An assignment *v* is a mapping that assigns to every variable $x_{i}\in X$ an element $v(x_{i})\in D_{i}$. An assignment *v* satisfies a constraint $((x_{i1},\ldots ,x_{ik}),H_{i})\in C$ iff $(v(x_{i1}),\ldots ,v(x_{ik}))\in H_{i}$. Finally, a solution is an assignment *v* to all variables such that all constraints are satisfied, denoted by $\left(v(x_{1}),\ldots ,v(x_{n})\right)$.

Finding a valid assignment of a CSP is in general NP-complete. Nevertheless, several programming libraries support constraint programming, like ECLiPSe, SWI Prolog, Gecode, JaCoP, Choco, Turtle (just to mention some of them) and allow for efficient implementations of CSPs. These constraint programming solvers make use of techniques like backtracking and local search.

Computing argumentation semantics via constraint programming has been addressed mainly by Amgoud and Devred [23] and Bistarelli and Santini [26–28], where the latter provide the system ConArg which is able to compute a wide range of semantics for abstract argumentation frameworks.

#### 3.2.1 Mappings of AFs to CSPs

Given an AF F=(A,R), the associated CSP (X,D,C) is specified as X=A and for each $a_{i}\in X$, $D_{i}=\{0,1\}$. The constraints are formulated depending on the specific semantics *σ*. For example, solutions that correspond to conflict-free sets can be obtained by defining a constraint for each pair of arguments *a* and *b* with (a,b)∈R, where the two variables may not be set to 1 at the same time. Here, the constraint is of the form ((a,b),((0,0),(0,1),(1,0))) which is equivalent to the cases when the propositional formula (a→¬b) evaluates to true.

In the following, we will use the notation from [23], because it reflects the similarities between the CSP approach and the reductions to propositional logic as outlined above.

For admissible semantics we get the following constraints.

(18) $$C_{\mathrm{adm}}=\left\{\left(a\to \underset{b:(b,a)\in R}{⋀}\neg b\right)\wedge \left(a\to \underset{b:(b,a)\in R}{⋀}\left(\underset{c:(c,b)\in R}{⋁}c\right)\right)|a\in A\right\}$$

The first part ensures conflict-free sets and the second part encodes the defense of arguments. Then, for an AF F=(A,R) and its associated admissible CSP $\left(X,D,C_{\mathrm{adm}}\right)$, $\left(v(x_{1}),\ldots ,v(x_{n})\right)$ is a solution of the CSP iff the set $\left\{x_{j},\ldots ,x_{k}\right\}$ s.t. $v(x_{i})=1$ is an admissible set in *F*.

Example 6 For the AF of Example 1 on Page 30 we obtain the following admissible CSP $\left(X,D,C_{\mathrm{adm}}\right)$. X=A, for each $a_{i}\in X$ we have $D_{i}=\{0,1\}$ and

$$C_{\mathrm{adm}}=\left\{(a\to ⊤)\wedge (a\to ⊤),(b\to \neg a\wedge \neg c)\wedge (b\to ⊥\wedge d),\;(c\to \neg b\wedge \neg d)\wedge \left(c\to (a\vee c)\wedge ⊥\right),(d\to ⊤)\wedge (d\to ⊤),\;(e\to \neg d\wedge \neg e)\wedge (e\to ⊥\vee d)\right\}.$$

This CSP has the following solutions: (0,0,0,0,0), (1,0,0,0,0), (0,0,0,1,0), (1,0,0,1,0) which correspond to the admissible sets of *F*, namely {},{a},{d} and {a,d}.

Most CSP solvers do not support subset maximization. Thus, for preferred semantics, the authors in [28] propose an approach that iteratively computes admissible/complete extensions and adds constraints to exclude certain sets, such that one finally obtains the preferred extensions.

#### 3.2.2 Reasoning problems

Similarly to the reductions to propositional logic, one can decide the following reasoning problems with CSPs, namely verification, skeptical and credulous reasoning. Furthermore, enumeration is usually supported by modern CSP solvers.

### 3.3 ASP-based approach

*Answer-set programming* (ASP, for short) [86,87], also known as A-Prolog [88,89], is a declarative problem solving paradigm, rooted in logic programming and non-monotonic reasoning. Due to continuous refinements over the last decade, answer-set solvers (e.g., [90,91]) nowadays not only support a rich language but also are capable of solving hard problems efficiently. Furthermore, the declarative approach of ASP results in readable and maintainable code (compared to C code, for instance), thus allowing to define the problems at hand in a natural way.

Solving problems in abstract argumentation via ASP has been studied by several authors (see [29] for a survey), including the approach proposed by Nieves et al. [74] where the program is re-computed for every input instance, Wakaki and Nitta [76] who use labeling-based semantics and the approach by Egly et al. [30] which follows extension-based semantics. Here, we focus on the latter, since this approach is put into practice by the ASPARTIX system which supports a wide range of different semantics and additionally offers a web front-end.

In the following, we first give a brief introduction to ASP. We then present how the computation of admissible and preferred extensions can be encoded in ASP. In order to obtain preferred extensions, it is necessary to check for subset-maximality of admissible sets. We sketch two approaches for this in ASP, one based directly on a certain *saturation technique* [92] (which is unfortunately hardly accessible for non-experts in ASP) and a second one which makes use of metasp encodings [93] (allowing to specify subset minimization via a single simple statement). Additionally, we discuss how reasoning problems can be specified.

#### 3.3.1 Answer-set programming

We give a brief overview of the syntax and semantics of disjunctive logic programs under the answer-set semantics [94]; for further background, see [95,91].

We fix a countable set U of *(domain) elements*, also called *constants*, and suppose a total order < over the domain elements. An *atom* is an expression $p(t_{1},\ldots ,t_{n})$, where *p* is a *predicate* of arity n≥0 and each $t_{i}$ is either a variable or an element from U. An atom is *ground* if it is free of variables. $B_{U}$ denotes the set of all ground atoms over U.

A *(disjunctive) rule r* with n≥0, m≥k≥0, n+m>0 is of the form

$$a_{1}\vee \cdots \vee a_{n}\leftarrow b_{1},\ldots ,b_{k},\mathrm{not}\;b_{k+1},\ldots ,\mathrm{not}\;b_{m}$$

where $a_{1},\ldots ,a_{n},b_{1},\ldots ,b_{m}$ are atoms, and “*not*” stands for *default negation*. An atom *a* is a positive literal, while *not* *a* is a default negated literal. The *head* of *r* is the set $H(r)=\{a_{1},\ldots ,a_{n}\}$ and the *body* of *r* is $B(r)=B^{+}(r)\cup B^{-}(r)$ with $B^{+}(r)=\{b_{1},\ldots ,b_{k}\}$ and $B^{-}(r)=\{b_{k+1},\ldots ,b_{m}\}$. A rule *r* is *normal* if n≤1 and a *constraint* if n=0. A rule *r* is *safe* if each variable in *r* occurs in $B^{+}(r)$. A rule *r* is *ground* if no variable occurs in *r*. A *fact* is a ground rule without disjunction and with an empty body. An *(input) database* is a set of facts. A program is a finite set of safe disjunctive rules. For a program *π* and an input database *D*, we often write π(D) instead of D∪π. If each rule in a program is normal (resp. ground), we call the program normal (resp. ground). Besides disjunctive and normal programs, we consider here the class of optimization programs, i.e., normal programs which additionally contain #*minimize* statements

(19) $$\#\mathrm{minimize}[l_{1}=w_{1}@J_{1},\ldots ,l_{k}=w_{k}@J_{k}]$$

where $l_{i}$ is a literal, $w_{i}$ an integer weight and $J_{i}$ an integer priority level.

For any program *π*, let $U_{\pi }$ be the set of all constants appearing in *π*. Gr(π) is the set of rules *rτ* obtained by applying, to each rule r∈π, all possible substitutions *τ* from the variables in *r* to elements of $U_{\pi }$. An *interpretation* $I\subseteq B_{U}$ *satisfies* a ground rule *r* iff H(r)∩I≠∅ whenever $B^{+}(r)\subseteq I$ and $B^{-}(r)\cap I=\emptyset$. *I* satisfies a ground program *π*, if each r∈π is satisfied by *I*. A non-ground rule *r* (resp., a program *π*) is satisfied by an interpretation *I* iff *I* satisfies all groundings of *r* (resp., Gr(π)). $I\subseteq B_{U}$ is an *answer set* of *π* iff it is a subset-minimal set satisfying the *Gelfond–Lifschitz reduct* $\pi^{I}=\{H(r)\leftarrow B^{+}(r)|I\cap B^{-}(r)=\emptyset ,r\in \mathrm{Gr}(\pi )\}$. For a program *π*, we denote the set of its answer sets by AS(π).

For semantics of optimization programs, we interpret the #*minimize* statement w.r.t. subset-inclusion: For any sets *X* and *Y* of atoms, we have $Y\subseteq_{J}^{w}X$, if for any weighted literal l=w@J occurring in (19), Y⊨l implies X⊨l. Then, *M* is a collection of relations of the form $\subseteq_{J}^{w}$ for priority levels *J* and weights *w*. A standard answer set (i.e., not taking the minimize statements into account) *Y* of *π dominates* a standard answer set *X* of *π* w.r.t. *M* if there are a priority level *J* and a weight *w* such that $X\subseteq_{J}^{w}Y$ does not hold for $\subseteq_{J}^{w}\;\in M$, while $Y\subseteq_{J^{\prime }}^{w^{\prime }}X$ holds for all $\subseteq_{J^{\prime }}^{w^{\prime }}\;\in M$ where $J^{\prime }\geq J$. Finally, a standard answer set *X* is an answer set of an optimization program *π* w.r.t. *M* if there is no standard answer set *Y* of *π* that dominates *X* w.r.t. *M* [93].

#### 3.3.2 Basic encodings

We now provide fixed queries for admissible and preferred extensions in such a way that the AF *F* is given as an input database $\overset{ˆ}{F}$ and the answer sets of the combined program $\pi_{e}(\overset{ˆ}{F})$ are in a certain one-to-one correspondence with the respective extensions, where e∈{adm,prf}. For an AF F=(A,R), we define

$$\overset{ˆ}{F}=\left\{\arg(a)|a\in A\right\}\cup \left\{\mathrm{att}(a,b)|(a,b)\in R\right\}.$$

We have to guess candidates for the selected type of extensions and then check whether a guessed candidate satisfies the corresponding conditions, where default negation is an appropriate concept to formulate such a guess within a query. In what follows, we use unary predicates in(⋅) and out(⋅) to perform a guess for a set S⊆A, where in(a) represents that a∈S.

Similar to Definition 7, we define the subsequent notion of correspondence which is relevant for our purposes.

Definition 8 Let $S\subseteq 2^{U}$ be a collection of sets of domain elements and let $I\subseteq 2_{U}^{B}$ be a collection of sets of ground atoms. We say that S and I correspond to each other, in symbols S≅I, iff

1. for each S∈S, there exists an I∈I, such that {a|in(a)∈I}=S;
2. for each I∈I, there exists an S∈S, such that {a|in(a)∈I}=S; and
3. |S|=|I|.

Next, we will successively introduce the rules our queries are composed of. Let F=(A,R) be an argumentation framework. The following program fragment guesses, when augmented by $\overset{ˆ}{F}$, any subset S⊆A and then checks whether the guess is conflict-free in *F*:

$$\pi_{\mathrm{cf}}=\{\mathrm{in}(X)\leftarrow \mathrm{not}\;\mathrm{out}(X),\arg(X);$$

πcf={out(X)←notin(X),arg(X);

πcf={←in(X),in(Y),att(X,Y)}.

The program module $\pi_{\mathrm{adm}}$ for the admissibility test is as follows:

$$\pi_{\mathrm{adm}}=\pi_{\mathrm{cf}}\cup \{\mathrm{defeated}(X)\leftarrow \mathrm{in}(Y),\mathrm{att}(Y,X);$$

πadm=←in(X),att(Y,X),notdefeated(Y)}.

For any AF F=(A,R), the admissible sets of *F* correspond to the answer sets of $\pi_{\mathrm{adm}}$ augmented by $\overset{ˆ}{F}$, i.e. $\mathrm{adm}(F)≅\mathrm{AS}(\pi_{\mathrm{adm}}(\overset{ˆ}{F}))$.

Sometimes we have to avoid the use of negation. This might either be the case for the saturation technique (described below), or if the problem can be encoded without a *guess & check* approach (e.g. for grounded semantics). Then, encodings typically rely on a form of loops where all domain elements are visited and it is checked whether a desired property holds for all elements visited so far. We will use this technique in our saturation-based encoding. For this purpose, an order < over the domain elements (usually provided by common ASP solvers) is used together with a few helper predicates defined in the program $\pi_{<}$ below; in fact, predicates lt/2, inf/1, succ/2 and sup⁡/1 denote lower than, infimum, successor and supremum of the order <. The predicates ninf/1, nsup/1 and nsucc/2 are used to derive that an element is not the infimum or the supremum, or respectively an element is not the successor of the other w.r.t. the order <.

$$\pi_{<}=\{\mathrm{lt}(X,Y)\leftarrow \arg(X),\arg(Y),X<Y;$$

π<={nsucc(X,Z)←lt(X,Y),lt(Y,Z);

π<={succ(X,Y)←lt(X,Y),notnsucc(X,Y);

π<={ninf(Y)←lt(X,Y);

π<={inf(X)←arg(X),notninf(X);

π<={nsup(X)←lt(X,Y);

π<={sup⁡(X)←arg(X),notnsup(X)}.

#### 3.3.3 Saturation encodings

To compute the preferred extensions of an argumentation framework, we will use the saturation technique as follows: Having computed an admissible set *S* (characterized via predicates in(⋅) and out(⋅) using encoding $\pi_{\mathrm{adm}}(\overset{ˆ}{F})$), we perform a second guess with new predicates, say inN(⋅) and outN(⋅), to represent a guess T⊃S. In order to check whether the first guess characterizes a preferred extension, we have to ensure that *no* guess of the second form (i.e., via inN(⋅) and outN(⋅)) characterizes an admissible set. The saturation module $\pi_{\mathrm{satpref}}$ looks as follows.

(20) $$\pi_{\mathrm{satpref}}=\{\mathrm{inN}(X)\vee \mathrm{outN}(X)\leftarrow \mathrm{out}(X);$$

(21) πsatpref={inN(X)←in(X);

(22) πsatpref={fail←eq;

(23) πsatpref={fail←inN(X),inN(Y),att(X,Y);

(24) πsatpref={fail←inN(X),outN(Y),att(Y,X),undefeated(Y);

(25) πsatpref={inN(X)←fail,arg(X);

(26) πsatpref={outN(X)←fail,arg(X);

(27) πsatpref={←notfail}.

Let us for the moment also assume that predicates eq (rule (22)) and undefeated(⋅) (rule (24)) are defined (we give the additional rules for those predicates below in the modules $\pi_{\mathrm{eq}}$ and $\pi_{\mathrm{undefeated}}$) and provide the following information:

• eq is derived if the guess *S* via in(⋅) and out(⋅) equals the second guess *T* via inN(⋅) and outN(⋅); in other words, eq is derived if S=T;
• undefeated(a) is derived if argument *a* is not defeated in *F* by the second guess *T*.

In the following, we discuss the functioning of $\pi_{\mathrm{satpref}}$ when conjoined with the program $\pi_{\mathrm{adm}}(\overset{ˆ}{F})$ for a given AF *F*. First, rule (20) guesses a set T⊆A as already discussed above. Rule (21) ensures that the new guess satisfies S⊆T.

The task of the rules (22)–(24) is to check whether the new guess *T* is a proper superset of *S* and characterizes an admissible set of the given AF *F*. If this is not the case, we derive the predicate fail. More specifically, we derive fail if either S=T (rule (22)); *T* is not conflict-free in *F* (rule (23)); or *T* contains an argument not defended by *T* in *F* (rule (24)). In other words, we have not derived fail if T⊃S and *T* is admissible in *F*. By definition, *S* then cannot be a preferred extension of *F*.

The remaining rules (25)–(27) saturate the guess in case fail was derived, and finally ensure that fail has to be in an answer set.

Let us illustrate now the behavior of $\pi_{\mathrm{satpref}}$ for two scenarios. First, suppose the first guess *S* (via predicates in(⋅) and out(⋅)) is a preferred extension of the given AF F=(A,R). Hence, for each T⊃S, *T* is not admissible. Consequently, *every* new guess *T* (via predicates inN(⋅) and outN(⋅)) derives fail. Thus, we have no interpretation without predicate fail that satisfies $\pi_{\mathrm{satpref}}$. However, the saturated interpretation, which contains fail and both inN(a) and outN(a) for each a∈A, does satisfy the program and also becomes an answer set of the program.

Now, suppose the first guess *S* (via predicates in(⋅) and out(⋅)) is an admissible but not a preferred extension of the given AF *F*. Then, there exists a set T⊃S, where *T* is admissible in *F*. If we consider the interpretation *I* characterizing *T* (i.e., we have inN(a)∈I, for each a∈T, and outN(a)∈I, for each a∈A∖T), then *I* does not contain fail and satisfies the rules (20)–(26). But this shows that we cannot have an answer set *J* which characterizes *S*. Due to rule (27) such an answer set *J* has to contain fail and by rules (25) and (26), *J* contains both inN(a) and outN(a) for each a∈A. Note that we thus have I⊂J (if *I* and *J* characterize the same initial guess *S*). Moreover, *I* satisfies the reduct of our program with respect to *J*. This can be seen by the fact that the only occurrence of default negation is in rule (27). In other words, there is an I⊂J satisfying the reduct and thus *J* cannot be an answer set. This, however, is the desired outcome, since the initial guess *S* characterized by *J* is not a preferred extension.

We still have to define the rules for the predicates eq and undefeated(⋅). Basically, these predicates would be easy to define, but as we have seen in the discussion above, default negation plays a central role in the saturation technique (recall the functioning of ←notfail). We therefore have to find encodings which suitably define the required predicates only with a limited use of negation. In fact, we are only allowed to have stratified negation in these modules. Thus, both predicates eq and undefeated(⋅) are computed via predicates eq_upto(⋅) (resp., undefeated_upto(⋅,⋅)) in the modules $\pi_{\mathrm{eq}}$ and $\pi_{\mathrm{undefeated}}$, which are defined as follows.

$$\pi_{\mathrm{eq}}=\{\mathrm{eq}\_\mathrm{upto}(X)\leftarrow \inf(X),\mathrm{in}(X),\mathrm{inN}(X);$$

πeq={eq_upto(X)←inf(X),out(X),outN(X);

πeq={eq_upto(X)←succ(Y,X),in(X),inN(X),eq_upto(Y);

πeq={eq_upto(X)←succ(Y,X),out(X),outN(X),eq_upto(Y);

πeq={eq←sup⁡(X),eq_upto(X)};

$$\pi_{\mathrm{undefeated}}=\{\mathrm{undefeated}\_\mathrm{upto}(X,Y)\leftarrow \inf(Y),\mathrm{outN}(X),\mathrm{outN}(Y);$$

πundefeated={undefeated_upto(X,Y)←inf(Y),outN(X),notatt(Y,X);

πundefeated={undefeated_upto(X,Y)←succ(Z,Y),undefeated_upto(X,Z),outN(Y);

πundefeated={undefeated_upto(X,Y)←succ(Z,Y),undefeated_upto(X,Z),notatt(Y,X);

πundefeated={undefeated(X)←sup⁡(Y),undefeated_upto(X,Y)}.

With these predicates at hand, we can now formally define the module for preferred extensions,

$$\pi_{\mathrm{prf}}=\pi_{\mathrm{adm}}\cup \pi_{<}\cup \pi_{\mathrm{eq}}\cup \pi_{\mathrm{undefeated}}\cup \pi_{\mathrm{satpref}}.$$

Then, for any AF *F*, the answer sets of $\pi_{\mathrm{prf}}(\overset{ˆ}{F})$ are in a one-to-one correspondence with the preferred extensions of *F*, i.e. $\mathrm{prf}(F)≅\mathrm{AS}(\pi_{\mathrm{prf}}(\overset{ˆ}{F}))$.

#### 3.3.4 metasp encodings

The following encodings for preferred semantics use the #*minimize* statement when evaluated with the subset-minimization semantics provided by metasp [93]. For our encodings we do not need prioritization and weights, therefore these are omitted (i.e., set to default) in the minimization statements. The minimization technique is realized through meta programming techniques, which themselves are answer-set programs. This operates as follows: The ASP encoding to solve is given to the grounder gringo^10^ which reifies the program, i.e., outputs a ground program consisting of facts, which represent the rules and facts of the original input encoding. The grounder is then again executed on this output with the meta programs that encode the optimization. Finally, claspD computes the answer sets. Note that here we use the version of clasp which supports disjunctive rules. Therefore, for a program *π* and an AF *F* we have the following program execution call.

**Figure:**
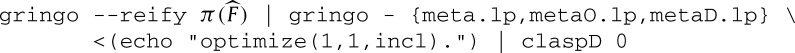

Here, meta.lp, metaO.lp and metaD.lp are the encodings for the minimization statement. The statement optimize(incl,1,1) indicates that we use subset inclusion for the optimization technique using priority and weight 1.

We now look at the encodings for preferred semantics which are easy to encode using the minimization statement of metasp. We only need the module $\pi_{\mathrm{adm}}$ and minimize the out/1 predicate. This in turn gives us the subset-maximal admissible sets which captures the definition of preferred semantics.

$$\pi_{\mathrm{prf}\_\mathrm{metasp}}=\pi_{\mathrm{adm}}\cup \left\{\#\mathrm{minimize}[\mathrm{out}]\right\}.$$

As a result we get that for any AF *F*, the answer sets of $\pi_{\mathrm{prf}\_\mathrm{metasp}}(\overset{ˆ}{F})$ are in a one-to-one correspondence with the preferred extensions of *F*, i.e. $\mathrm{prf}(F)≅\mathrm{AS}(\pi_{\mathrm{prf}\_\mathrm{metasp}}(\overset{ˆ}{F}))$.

#### 3.3.5 Reasoning problems

As with other reduction-based approaches, the types of reasoning available depend on the ASP solver. Many of them feature enumeration of all solutions, as well as counting and also credulous and skeptical reasoning. For the metasp variant, the meta encodings can be augmented with constraints to achieve credulous and skeptical reasoning. Usually, when one uses an ASP program, like $\pi_{\mathrm{prf}}$ for preferred semantics, for an AF *F*, the ASP solver returns all answer sets of the program which correspond to all preferred extensions of *F*. For credulous or skeptical acceptance, one uses the respective reasoning problems of the ASP solver, which are sometimes referred to as *brave* or *cautious* reasoning. Note that the concrete usage depends on the particular solver.

We exemplify this option for the solver dlv. To compute all arguments which are skeptically accepted under preferred semantics for a given AF *F*, we invoke

$$\text{dlv -cautious}\;\pi_{\mathrm{prf}}(\overset{ˆ}{F})\cup \left\{\mathrm{in}(X)?\right\}$$

### 3.4 Further reduction-based approaches

In the following, we summarize further approaches for reduction-based methods.

#### 3.4.1 SAT extensions

Several extensions to the prototypical problem of SAT have been developed. Two interesting approaches include so-called *minimal correction sets* (MCSes) [96] and the *backbone* [97] of a Boolean formula in CNF. The former consists of a minimal subset of the clauses of an unsatisfiable formula, such that if we remove these clauses the resulting formula becomes satisfiable. A backbone of a satisfiable formula is simply the set of literals it logically entails. These concepts can be used to answer several reasoning problems for AFs and optimize existing algorithms. In [98] MCSes are applied to solve reasoning problems for semi-stable semantics by encoding the range of a set as satisfied clauses, and techniques for computing backbones are utilized to enhance the efficiency of the algorithms.

#### 3.4.2 Equational approaches

Equational approaches for abstract argumentation map the given reasoning problem at hand to a set of equations. Solutions of such equations then directly represent solutions of the original problems. One such approach was proposed by Gabbay [32,77]. Here, one receives a system of equations where each argument is represented by a distinct variable with a domain of real numbers in the interval [0,1]. Solutions to these systems of equations assign to each variable a number from the domain. The variable assignment corresponds to a labeling. If the variable of an argument *a* is mapped to 1, *a* is in, if it is mapped to 0 then it is out and undecided otherwise. This allows for easy identification of the labelings by inspecting the variable assignments. For instance, to compute complete labelings in this way we can consider the equation $f(a)=1-\max(f(x_{1}),\ldots ,f(x_{n}))$ where $\left\{x_{1},\ldots ,x_{n}\right\}$ are the attackers of *a*. If all attackers of *a* are mapped to 0, then the value of *a* will be 1. If at least one of the attackers is “in”, i.e. it is mapped to 1, then f(a)=0. Otherwise, if all attackers are undecided with $1<f(x_{i})<0$ for each 1≤i≤n, then *a* will be undecided.

Another work on equational approaches is by Osorio et al. [99,100]. They encode preferred and semi-stable semantics as integer programs and then use the solver Xpress^11^ to compute the extensions.

#### 3.4.3 Monadic second order logic

A reduction approach going beyond pure propositional logic is to encode the reasoning problems in monadic second order logic (MSO). In this expressive predicate logic we may quantify over variables and unary predicates. Given such an MSO formula and an interpretation *I*, the task is to check whether *I* is a model of the formula. In [33] the authors encode several reasoning problems for AFs into an MSO formula *φ*. A given AF is then transformed into an interpretation *I* and one decides the reasoning problem by testing whether *I* is a model of *φ*. In this work certain building blocks for such encodings are introduced, which facilitate straightforward reductions of the different semantics to MSO. While the first MSO-encodings for abstract argumentation [101,14] were introduced to obtain complexity-theoretic results in terms of tree-width, the advent of efficient systems for MSO [102,103] turns MSO-encodings into an interesting alternative to implement abstract argumentation via the reduction method.

## 4 Direct approaches

In the previous section, we exhaustively discussed different reduction-based approaches for implementing abstract argumentation. But what about implementing procedures for abstract argumentation from scratch? While such an approach definitely requires more effort in implementation, it allows to access the framework directly, without having the overhead of transformation (and as a result a potential loss of structural information). Even more important, compared to the reduction approach, direct algorithms allow for an easy incorporation of short-cuts that are specific for the argumentation domain.

In the reduction-based approach the distinction between computing all extensions and performing specific reasoning tasks is often delegated to the reasoner of the target formalism and thus can be neglected. When using direct approaches we have to take care (and advantage) of specific reasoning problems ourselves. Hence, in this section we will distinguish more explicitly between algorithms for enumerating all extensions and, for instance, algorithms that are specially tailored for computing “witnesses” for certain queries.

Nowadays, the most successful approaches for direct algorithms can be categorized in three groups. First, there are so called *labeling-based algorithms* [104,35,38], which build on alternative characterizations for argumentation semantics using certain labeling functions of arguments. Second, we consider dialectical argument games, i.e., games played by two players alternating their arguments and where winning strategies ultimately characterize the acceptance status of an argument. Finally, there are *dynamic programming algorithms*, which are based on graph decompositions and results from (parameterized) complexity analysis. In the following, we present each of these approaches in detail.

### 4.1 Labeling-based algorithms

The class of labeling-based algorithms builds on the concept of argument labelings, with probably the most prominent variant being the 3-valued labelings due to Caminada and Gabbay [69]. For the formal definitions of complete and preferred labelings we refer to Section 2 (Definitions 4 & 5).

First labeling-based algorithms have been proposed in [34]; many further materializations of this concept can be found in the literature (see, e.g., [35,38,36]). The central observation underlying all these approaches is the following: Whenever one fixes the label of one argument this has immediate implications for the possible labels of the neighbors of this argument. For instance, if we are interested in complete labelings and label an argument *a* with *in* then all neighbors of *a* must be labeled *out*.

In what follows, we focus on labeling-based algorithms for preferred semantics and distinguish between two classes: (i) algorithms which aim to enumerate all preferred extensions of a given AF; and (ii) algorithms that are tailored to perform specific reasoning tasks like skeptical and credulous reasoning.

#### 4.1.1 Enumerating extensions

For enumerating extensions one can, in principle, simply enumerate all possible sets and check whether they are extensions. In general this is of course a quite inefficient approach. Therefore, labeling-based algorithms typically use a particular backtracking strategy to enumerate possible labelings, fixing the label of one argument in each step. In addition to the simple backtracking strategy, in each step the information of the new label is propagated to the neighbors of the argument. The different approaches to labeling-based algorithms have their own strategy for selecting the next arguments to be labeled as well as for the rules they apply for propagating labels. Algorithm 3 is an example for a labeling-based algorithm for computing preferred labelings in the spirit of [35].

The main idea of Algorithm 3 is to start with the labeling that marks all arguments with *in* (the set containing all arguments) and to re-label arguments to either *out* or *undec* until the set becomes admissible. This strategy prevents the algorithm from considering all the (relatively small) admissible sets as candidates for preferred extension like other algorithms do (compare Algorithm 4).

Let us explain Algorithm 3 in more detail. When applying the algorithm to an AF F=(A,R), it first initializes the labeling L such that each argument is labeled with *in*, i.e., $L_{\mathrm{in}}=A$, and the set $S_{L}$ of candidate solutions only contains the labeling (∅,∅,A), corresponding to the empty set. Then, in each step the algorithm picks an argument *a* which is labeled *in* but is not defended, i.e., there is an attacker that is not labeled *out*, and relabels it. We call such a relabel step a transition step. In Algorithm 3 a transition step is due to the following rules. First, the argument *a* is labeled to *out* and then all arguments $y\in {\left\{a\right\}}^{+}$ (*a* and all arguments attacked by *a*) are checked for being valid labeled *out*, i.e., in case *y* is neither labeled *in* nor is attacked by an argument labeled *in*, we change the label of *y* to *undec*. In [35] it is shown that each preferred extension can be obtained from the initial labeling that labels each argument to *in* by a finite sequence of such transition steps and further that each terminated sequence (which is indeed finite) corresponds to an admissible set.

This simple algorithm has several weaknesses which have been addressed in the literature. First, consider line 5 of Algorithm 3. For each $a\in L_{\mathrm{in}}$ s.t. $\exists b\notin L_{\mathrm{out}}:(b,a)\in R$ one starts a transition and then recursively calls the procedure. This causes the branching in the search procedure and thus we want to minimize the number of arguments to be considered here. To this end, [35] introduces a notion of so-called super-illegal arguments which form a subset of the above mentioned arguments and can be relabeled first without branching in the algorithm. That is, in case there is at least one super-illegal argument the algorithm first considers all of them (in arbitrary order) before branching among the other arguments. However, even with this improvement, it can happen that several branches of the algorithm may produce the same candidate extension. For instance, consider the AF F=({a,b,c},{(a,b),(b,a),(b,c),(c,b)}). The preferred labeling 〈{b},{a,c},∅〉 will be produced by two branches of the algorithm, by the branch choosing *a* in the first step (and assign *out* to *a*) and then choosing *c* in the second step (and assigning *out* as well), but also by the branch selecting *c* first and then *a* in the second step. As such duplicates are indeed a waste of computational resources, this is a weak point. Other algorithms [34,36] avoid such duplicates as they use a different strategy to branch in the search space (see, for instance, Algorithm 4).

Next, consider lines 16–23 in the algorithm. This part ensures the ⊆-maximality of the labelings in $S_{L}$. As the set $S_{L}$ can be of exponential size (even if the number of preferred labelings is small) testing whether a new candidate is ⊆-maximal and updating the set $S_{L}$ is costly. Hence, alternative approaches to deal with ⊆-maximality have been proposed. Firstly, [34] used a criterion for maximality that does not make use of the other extensions explicitly. Instead, it exploits the observation that a complete labeling L is a preferred labeling iff there is no subset *S* of $L_{\mathrm{undec}}$ such that the set $L_{\mathrm{in}}\cup S$ is admissible. In particular, for candidate labelings where all arguments are labeled either *in* or *out*, this avoids an explicit check of maximality (such labelings correspond to stable extensions). Secondly, in [36] the authors provide a smart traversal of the search space such that one can avoid deleting sets from $S_{L}$, i.e., in each step one can decide whether the current candidate is preferred or not, by only using previously computed preferred labelings (see Algorithm 4).

Let us thus have a closer look on Algorithm 4 next. This algorithm for preferred semantics follows the work of [34] and [36]. The main difference to Algorithm 3 is the way the search space is explored. Starting with all arguments being unlabeled in each step the algorithm chooses one (unlabeled) argument and branches between the possible labels for this argument. Once a label is chosen it is never changed again and thus no labeling can be produced twice. Another apparent difference is that the algorithm uses four labels instead of just three labels. We denote such a four valued labeling L as quadruple $\langle L_{\mathrm{in}},L_{\mathrm{out}},L_{\mathrm{att}},L_{\mathrm{undec}}\rangle$.^12^ The intuition behind the labels *in* and *out* is the same as for three valued labelings, while arguments which attack an *in* labeled argument, but are not attacked by such an argument, are labeled *att*. Finally, arguments which are labeled *undec* have no conflict with *in* labeled arguments.

This algorithm iterates over all admissible sets and tests whether they are ⊆-maximal. As for each argument *a* the algorithm first tries to add an argument to $L_{\mathrm{in}}$ before considering the variant without *a*, we can be sure that supersets are always considered first. Hence, we never have to remove a labeling from the set $S_{L}$. The pitfall of Algorithm 4 is the potential exponential number of admissible labelings (even for a small number of preferred extensions) which are all considered by the algorithm.

Let us briefly compare Algorithm 3 and Algorithm 4 on two extreme cases: (i) the AF $F_{1}=(A,A\times A)$ with the total attack relation and (ii) the AF $F_{2}=(A,\emptyset )$ with the empty attack relation. In $F_{1}$ the empty set is the only admissible set and thus also the only preferred extension. As there is just one admissible set, Algorithm 4 never branches and thus terminates after a linear number of steps. However Algorithm 3 has to update all arguments to *undec*. As this can be done in an arbitrary order we have *n*! many branches producing the same extension. For $F_{2}$ there is just one preferred extension but Algorithm 4 considers all $2^{\left|A\right|}$ admissible sets. In contrast, Algorithm 3 terminates immediately. As different labeling-based algorithms behave good on different kind of argumentation frameworks, empirical evaluations are an important issue. A first step in that direction is done in the work of Nofal et al. [105,36,37] where the performance of different labeling-based algorithms for preferred semantics is compared on randomly generated AFs.

Here we have only considered the case of preferred semantics, but for most of the semantics labeling-based algorithms have been proposed in the literature: an algorithm for grounded semantics is given in [35]; an algorithm for admissible labelings can be easily obtained from Algorithm 4 (by dropping the ⊆-maximality test in line 14); for complete semantics one can adapt Algorithm 3; for stable semantics, see [35]; algorithms for semi-stable and stage semantics can be found in [104,106,35]. Recently, [105] studied improved algorithms for enumerating grounded, complete, stable, semi-stable and stage semantics. Labeling-based algorithms are implemented in the ArguLab system [107] as well as in ArgTools [36] (see also Section 5.1).

#### 4.1.2 Reasoning problems

Having an algorithm for enumerating all extensions of an AF at hand, one can immediately use them to answer reasoning problems by simply testing each extension for the queried argument. However, this is probably not the most efficient way. Given that we are only interested in the acceptance of a certain argument, we might directly try to produce a witness (or counter-example) for this argument instead of computing all extensions. In this section we discuss dedicated algorithms for reasoning problems. As an example, we review the work of Verheij [38], a credulous acceptance algorithm for preferred semantics, which is implemented in the *CompArg* system (see Section 5.1).

The idea behind the algorithm is that we start with the argument (or the set of arguments) for which we test credulous acceptance and iteratively add arguments to defend all arguments in our sets. The outlined Algorithm 5 starts with labeling the queried argument with *in* and all the other arguments with *undec*. Then, it iterates the following two steps. First, check whether the set $L_{\mathrm{in}}$ is conflict-free and if so label all arguments attacking $L_{\mathrm{in}}$ with *out*. Otherwise terminate the branch of the algorithm. In the second step, we pick for each argument *a* which is labeled *out* but not attacked by an argument labeled *in*, an *undec* labeled attacker *b* of *a* and label it with *in*. In case there are several such arguments, we start a new branch of the algorithm for each choice. If no such argument exists we terminate the branch of the algorithm. We stop a branch of the algorithm as soon as no more changes to labelings are made. In that case we have reached an admissible labeling acting as proof for the credulous acceptance of the queried argument.

We give a few more comments for Algorithm 5. In contrast to the previous algorithm, we store several partial extensions (partial proofs) at the same time and also terminate as soon as we have found an admissible set. In line 1 of the algorithm one has to decide whether to use a queue or a stack for storing these partial proofs. The choice determines the search strategy in the space of partial proofs: The former would give a breadth-first search (as suggested in [38]) while the latter yields a depth-first search.

Next, consider the sets $L^{\prime }$ in line 11. These are simply the sets where for each argument $a\in L_{\mathrm{out}}\setminus L_{\mathrm{in}}^{+}$ we pick one argument *b* attacking *a* and add *b* to $L_{\mathrm{in}}^{+}$. However, in each step there might be exponentially many such sets $L^{\prime }$. In case there is no such set, we know the partial proof cannot be expanded to a proof and we can close this branch of the search tree. Moreover, it can happen that we consider the same partial proof twice, and thus it might be a good idea to store already considered partial proofs.

Finally, let us mention that beside the work of Verheij [38], Doutre and Mengin [34] suggest to start from an enumeration algorithm similar to Algorithm 4 but employing several shortcuts for credulous and skeptical reasoning.

### 4.2 Dialectical proof-procedures (dialogue games)

A popular approach for obtaining proof procedures for abstract argumentation is based on so called dialogue games (see, e.g., [35,39]). Such games are played by two players, the proponent (Pro) and the opponent (Opp), on a given argumentation framework. The proponent and opponent alternate in raising arguments of the AF attacking arguments previously raised by the other player (according to certain rules). A player loses the game if she cannot raise any argument. Typically, an argument *a* being accepted is equivalent to one player having a winning strategy in the dialogue game when started with *a*. However, in certain dialogue games it suffices that the proponent wins one of the possible dialogues starting with argument *a* to guarantee the acceptance of *a* [108]. By their nature, dialogue games are typically dedicated to a specific reasoning problem, but sometimes they can also be used to actually compute extensions.

Such algorithms are implemented in the *Dungine* system [109], in the *Dung-O-Matic* Java library (see Section 5.1) and also are used in Visser's Epistemic and Practical Reasoner,^13^ a tool for argumentation with propositional languages (however, it is not a dedicated tool for abstract argumentation).

#### 4.2.1 Games for grounded and preferred semantics

In the following, we consider games for grounded semantics and for credulous acceptance with preferred semantics, both borrowed from [35]. In both cases the game is started by Pro raising the argument in question, and then Pro and Opp alternately raise an argument attacking the previous argument in the dialogue. Finally, a dialogue is won by the player making the last move, i.e., the player forcing the dialogue into a situation where the other player has no legal move left. The dialogue games correspond to our reasoning problems in the sense that Pro has a winning strategy in the game iff the argument is accepted. The games for the different semantics and reasoning problems differ in the allowed moves for the players, where typically Pro and Opp have different rule sets for legal moves.

##### A game for grounded semantics

First, consider a game that provides, given an AF F=(A,R) and argument a∈A, a proof whether *a* is contained in the grounded extension of *F*. The game is given by the following rules of allowed moves of each player.

*Legal moves:*

• For Pro: Any argument *y* that (i) attacks the last argument raised by Opp and (ii) is conflict-free with all arguments previously raised by Pro.
• For Opp: Any argument *y* that attacks the last argument raised by Pro.

One can easily show that *a* is in the grounded extension iff Pro has a winning strategy for the above game [35].

Example 7 Consider the AF from Example 1. The grounded extension is {a,d}. Now, if Pro starts a dialogue game with raising argument *a*, then, as *a* is not attacked at all, Opp has no legal move to reply. Hence, Pro has a winning strategy which reflects the fact that *a* is in the grounded extension. Next, consider Pro starts a dialogue game with raising argument *b* (an argument not in the grounded extension). Then, Opp has two legal moves, either raising *a* or *c*. In the first case Opp wins the game as *a* is not attacked at all and thus Pro has no legal moves. Hence, Pro has no winning strategy when starting with *b*.

##### A game for credulous preferred acceptance

Now let us consider a game that allows us, given an AF F=(A,R) and argument a∈A, to prove whether *a* is contained in some preferred extension of *F* (or equivalently in some admissible set). The following game is quite similar to the game for grounded semantics, the only difference being that Opp is not allowed to repeat its moves. Restricting the legal moves of Opp makes it easier to have a winning strategy for Pro.

*Legal moves:*

• For Pro: Any argument *y* that (i) attacks the last argument raised by Opp and (ii) is conflict-free with all arguments previously raised by Pro.
• For Opp: Any argument *y* that (i) attacks the last argument raised by Pro and (ii) was not previously used by Opp.

It can be shown that Pro has a winning strategy for the above game iff the argument *a* is in an admissible set. The latter is well known to be equivalent to argument *a* being credulously accepted with preferred semantics [35].

Example 8 Consider the very simple AF F=({a,b},{(a,b),(b,a)}) with the admissible sets {}, {a} and {b}. Now, let us test for the credulous acceptance of *a*, i.e., Pro starts the game with raising *a*. Then, the only option of Opp is to use *b*, Pro can use *a* again to defeat *b*. Now Opp has no legal move left, as it cannot use *b* again. Hence, Pro has a winning strategy for *a*. Notice that in the grounded game Pro and Opp would loop forever with raising *a* and *b*.

The critical reader might observe that such dialogue games are indeed no algorithms. However, it is more or less straight forward to build algorithms out of such games, using search procedures in the strategy space of these games, branching along the possible moves (see [39,110]). The resulting algorithms are indeed of a similar type as the previously discussed labeling-based algorithms.

### 4.3 Dynamic-programming based approach

As discussed in Section 2 most of the reasoning problems in abstract argumentation were shown to be computationally intractable, i.e., NP-hard or even harder. Hence, there is a lot of work on first classifying the exact (sources of) complexity of these problems and second on identifying problem classes that can be solved efficiently. Here we discuss algorithms based on ideas from parameterized complexity theory. The main observation is that binding a certain problem parameter to a fixed constant makes many of the intractable problems tractable. This property is referred to as *fixed-parameter tractability* (FPT) (see, e.g., [111]). The complexity class FPT consists of problems that can be computed in $f(k)\cdot n^{O(1)}$ where *f* is a function that depends on the problem parameter *k*, and *n* is the input size.

One important parameter for graph problems is the *tree-width* of a graph that is defined along so-called tree decompositions. Intuitively, the tree-width measures the tree-likeliness of a graph, in particular connected graphs of tree-width 1 are exactly trees. AFs can be seen as directed graphs and therefore the parameter tree-width can be directly applied to them. Indeed, many argumentation problems have been shown to be solvable in linear time for AFs of bounded tree-width [41,33].

In this section we present a dynamic-programming based approach for abstract argumentation that is defined on tree decompositions. First introduced in [112], this approach especially aimed at the development of efficient algorithms that turn complexity-theoretic results into practice. The algorithms from [112] are capable of solving credulous and skeptical reasoning problems under admissible and preferred semantics. Later, this approach was extended to work with stable and complete semantics [42]. Further fixed-parameter tractability results were obtained for AFs with bounded clique-with [113] and in the work on backdoor sets for argumentation [114]. Negative results for other graph parameters like bounded cycle-rank, directed path-width, and Kelly-width can be found in [40].

The approach presented here is put into practice in the dynPARTIX tool [42,115] as well as in the D-FLAT system [102,116]. While the former is a dedicated tool for argumentation (see also Section 5), the latter is a general framework that allows one to declaratively specify algorithms on tree decompositions by means of ASP.

In the following, we first introduce tree decompositions. We then present the general idea behind the dynamic-programming algorithms and provide an example based on admissible semantics. Finally, we discuss how the basic algorithm that enumerates all admissible sets can be adapted for other reasoning problems.

#### 4.3.1 Tree decompositions

A tree decomposition [117] is a mapping of a graph to a tree, defined as follows.

Definition 9 A *tree decomposition* of an undirected graph G=(V,E) is a pair (T,X) where $T=(V_{T},E_{T})$ is a tree, with vertices $V_{T}$ and edges $E_{T}$, and $X:V_{T}\to 2^{V}$ is a function that assigns to every vertex $t\in V_{T}$ of the tree a so-called bag, i.e. a set $X_{t}\subseteq V$ of vertices from the original graph. These sets of vertices ${\left(X_{t}\right)}_{t\in V_{T}}$ have to satisfy the following conditions:

(i) ${⋃}_{t\in V_{T}}X_{t}=V$
(ii) $(v_{i},v_{j})\in E\Rightarrow \exists t\in V_{T}:\{v_{i},v_{j}\}\subseteq X_{t}$
(iii) $v\in X_{t_{1}}\wedge v\in X_{t_{2}}\wedge t_{3}\in \mathrm{path}(t_{1},t_{2})\Rightarrow v\in X_{t_{3}}$

A set $X_{t}$ is also called the *bag* for the vertex *t*.

Conditions (i) and (ii) guarantee that no information of the original graph is lost, i.e., all vertices have to appear in at least one bag $X_{t}$ and connected vertices have to appear together in some bag. Condition (iii) is the connectedness condition, ensuring that all bags containing the same vertex are connected in T. In general a graph may have multiple tree decompositions. The parameter tree-width is defined on the “best” tree decompositions one can get for a graph.

Definition 10 The *width* of a tree decomposition (T,X) is defined as $\max(|X_{t\in V_{t}}|)-1$. The *tree-width* of a graph *G* is the minimum width of all possible tree decompositions of *G*.

Here, we will only consider *normalized* tree decompositions, which can be easily obtained from standard tree-decompositions [118]. Normalized tree decompositions comply with Definition 9, but only consist of the following four different node types:

1. *JOIN* node: A node *t* which has two children $t^{\prime }$ and $t^{\prime \prime }$, $X_{t}=X_{t^{\prime }}=X_{t^{\prime \prime }}$.
2. *INTRODUCTION* node: A node *t* having exactly one child $t^{\prime }$ s.t. $|X_{t}|=|X_{t^{\prime }}|+1$ and $X_{t^{\prime }}\subset X_{t}$.
3. *REMOVAL* node: A node *t* having exactly one child $t^{\prime }$ s.t. $|X_{t}|=|X_{t^{\prime }}|-1$ and $X_{t}\subset X_{t^{\prime }}$.
4. *LEAF* node: A node *t* that has no child nodes.

**Fig. 3:**
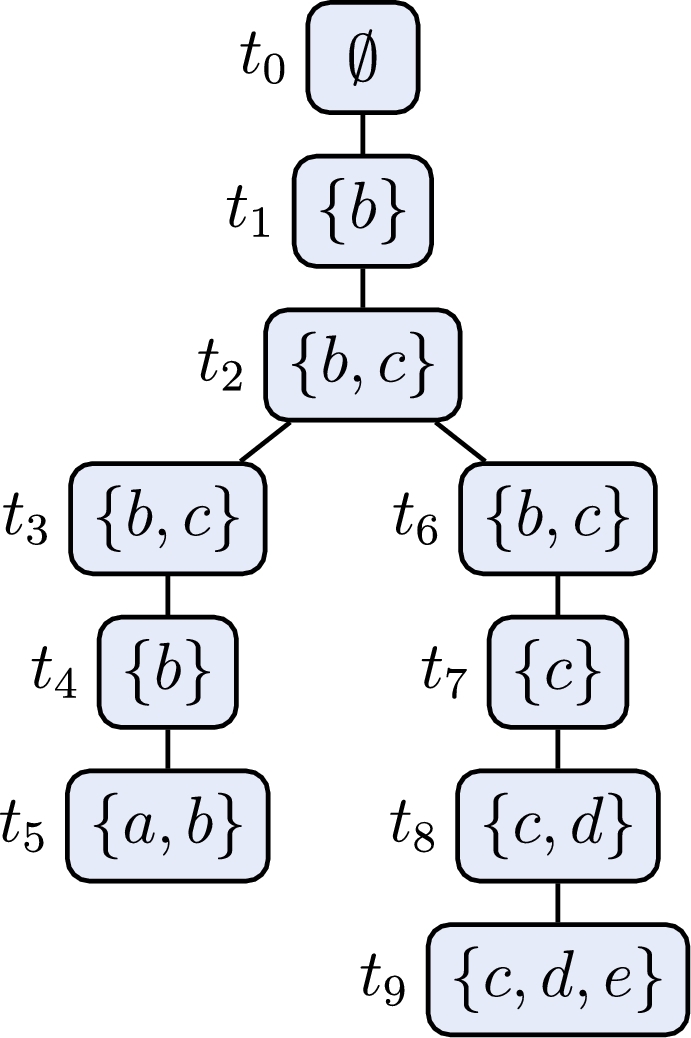
Normalized tree decomposition.

Additionally, we assume that for root node *r* of the normalized tree-decomposition, we have $X_{r}=\emptyset$. Note that tree decompositions are defined on undirected graphs. We relate AFs (see Definition 1) to tree decompositions by defining that a tree decomposition of an AF F=(A,R) is a tree decomposition of an undirected graph $G=(A,R^{\prime })$ where *A* are the arguments of the AF and $R^{\prime }$ are the edges *R* without orientation. In Fig. 3 one possible normalized tree decomposition of our example AF from Fig. 1 is given. The width of this tree decomposition is 2. Note that the computation of an optimal tree decomposition (w.r.t. width) is known to be an NP-complete problem [119]. However, the problem is fixed parameter tractable w.r.t. treewidth [120]. Thus, for AFs with small treewidth an optimal tree decomposition can be computed efficiently. Moreover, there exist several heuristic-based algorithms that provide “good” tree decompositions in polynomial time (see, e.g., [121,15,122]).

#### 4.3.2 Dynamic programming

In the following, we present a dynamic-programming algorithm for computing admissible sets (and deciding credulous acceptance of arguments) as proposed in [112]. Algorithms for other semantics and reasoning problems can be defined similarly.

In a nutshell, the idea of dynamic programming as used here is as follows. First, a tree decomposition of the given problem instance (AF) is constructed. The tree of that decomposition is then traversed in bottom-up order. Due to the definition of tree decompositions it is possible to only work on the information that is locally available in the bags when traversing the tree. In every step we compute so-called colorings, a data structure to represent (partial) solutions for our problem. These colorings are computed based on the arguments contained in the bag of the current node as well as colorings from the child node(s). The idea of dynamic programming is hereby realized as follows: If we encounter a REMOVAL node during bottom-up traversal then we can exploit the fact that the “removed” node will never reappear in another bag later on during the traversal. We can therefore discard information for (partial) solutions in case it contains a removed argument that does not fulfill the properties of our semantics. The solutions for the whole input instance can be obtained by a final computation step in the root node.

##### Sub-frameworks

**Fig. 4:**
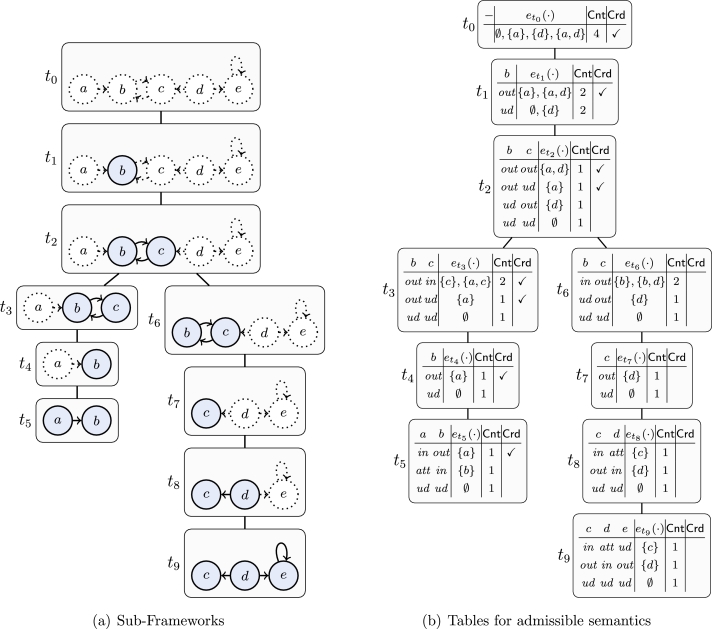
Normalized tree decomposition in action.

Towards the dynamic programming algorithm we have to introduce some notions that underlie the approach. First, for a tree decomposition (T,X) of an AF *F*, let t∈T. For a sub-tree of T that is rooted in *t* we define $X_{\geq t}$ as the union of all bags within this sub-tree, i.e., $X_{\geq t}$ contains all arguments of this sub-tree. For instance in the tree decomposition from Fig. 3 we have $X_{\geq t_{3}}=X_{t_{3}}\cup X_{t_{2}}\cup X_{t_{1}}=\{a,b,c\}$. Additionally, $X_{>t}$ denotes $X_{\geq t}\setminus X_{t}$, i.e., all arguments from the bags in the sub-tree excluding the arguments from the bag of *t* (even if they appear in another bag). Regarding the example we have $X_{>t_{3}}=(X_{t_{2}}\cup X_{t_{1}})\setminus X_{t_{3}}=\{a\}$. Furthermore, for a t∈T the *sub-framework in t*, denoted by $F_{t}$, consists of all arguments $x\in X_{t}$ and the attack relations $\left(x_{1},x_{2}\right)$ where $x_{1},x_{2}\in X_{t}$ and $(x_{1},x_{2})\in R$. The *sub-framework induced by the sub-tree rooted in t*, denoted by $F_{\geq t}$, consists of all arguments $x\in X_{\geq t}$ and the attack relations $\left(x_{1},x_{2}\right)$ where $x_{1},x_{2}\in X_{\geq t}$ and $(x_{1},x_{2})\in R$. Consider the tree decomposition given in Fig. 4(a). For each node *t*, the arguments that are contained in bag $X_{t}$ are marked with solid cycles. The sub-framework $F_{t}$ consists of the arguments in solid cycles and all solid attack arrows. In combination with the dotted parts we obtain the induced sub-frameworks $F_{\geq t}$.

##### Restricted sets

The idea is now to analyze the (sub)-framework $F_{\geq t}$ for every node *t* during our traversal. $X_{>t}$ denotes all arguments that were already removed from the bags of the sub-tree rooted at *t*. Hence, these arguments are already completely processed by the algorithm and we can define $X_{>t}$*-restricted admissible sets*. We distinguish between attacks from arguments in $X_{>t}$ and $X_{t}$. While attacks from arguments $X_{t}$ might be counter attacked by arguments appearing later, i.e., somewhere above in the tree decomposition, this cannot happen for arguments in $X_{>t}$. Thus, we define an $X_{>t}$-restricted admissible set *S* for a sub-framework $F_{\geq t}$ such that first *S* has to be conflict-free and second it has to defend itself against the arguments in $X_{>t}\setminus S$. So the $X_{>t}$-restricted admissible sets for a sub-framework $F_{\geq t}$ are all the sets that might become admissible in a framework $F_{\geq t^{\prime }}$ for some node $t^{\prime }$ above in the tree decomposition.

##### Colorings

In order to represent the information that is computed within each node during traversal we need an appropriate data structure. We define so-called *colorings* that allow us to store information of relationships between arguments in $X_{\geq t}$ solely by assigning colors to arguments in $X_{t}$. For admissible semantics, this is described by a function $C:X_{t}\to \{\mathrm{in},\mathrm{out},\mathrm{att},\mathrm{ud}\}$. Intuitively, *in* denotes that an argument is contained in the set *S* of selected arguments, *out* describes that it is outside the set because it is attacked by *S*, *att* means that the argument attacks *S* but is not attacked by *S* and *ud* describes that the status is undecided (it is neither attacked nor attacks *S*). Notice that this definition is quite close to the definition of the labelings used in Algorithm 4. The main difference is that a labeling labels the whole set of arguments while colorings are only applied to a small part of the set of arguments, even if other parts have already been considered.^14^ Towards a more concise notion, for a coloring *C*, the set ${\left[C\right]}_{\mathrm{in}}$ denotes all arguments that are colored with *in*.

We are in particular interested in colorings corresponding to at least one restricted admissible set, so-called *valid colorings*. Given a coloring *C* for node *t*, we define the *extensions of C*, $e_{t}(C)$, as the collection of $X_{>t}$-restricted admissible sets *S* for $F_{\geq t}$ that satisfy the following conditions for each $a\in X_{t}$:

$$\begin{array}{cc} C(a)=\mathrm{in} & \;\text{iff }a\in S\\ C(a)=\mathrm{out} & \;\text{iff }S{↣}^{R}a\\ C(a)=\mathrm{att} & \;\text{iff }S{↣̸}^{R}a\text{ and }a{↣}^{R}S\\ C(a)=\mathrm{ud} & \;\text{iff }S{↣̸}^{R}a\text{ and }a{↣̸}^{R}S \end{array}$$

If $e_{t}(C)\neq \emptyset$, *C* is called a *valid coloring* for *t*.

##### Goal

Our overall goal is to compute admissible sets of an AF. The tree decomposition is traversed in bottom-up order. In each node we use our data structure of colorings and compute all valid colorings *C* for every node *t*. As shown in [112], there exists a one-to-one mapping between the extensions of *C*, $e_{t}(C)$, and the $X_{>t}$-restricted admissible sets for $F_{\geq t}$. Moreover, we assume that the root node *r* has an empty bag of arguments. Hence, by computing the valid colorings *C* for *r* we obtain the $X_{>r}$-restricted admissible sets for $F_{\geq r}$. As $X_{>r}=A$ these correspond to the admissible sets for our original AF instance.

##### Node operations

In order to achieve tractability we have to compute valid colorings in bottom-up order without explicit computation of the corresponding restricted admissible sets $e_{t}(C)$. Hence, we define operations for the computation of valid colorings which are applied recursively on the colorings computed at the child node(s). Detailed arguments for the correctness of these operations are given in [40], we shall just sketch the intuition behind them here.

Let t∈T be a node and $t^{\prime }$ and $t^{\prime \prime }$ be its children, if they exist. Depending on the node type of *t* we apply the following operations:

LEAF node: Here we have $F_{t}=F_{\geq t}$ and thus the restricted admissible sets are just the conflict-free sets. So we compute the conflict-free sets of $F_{t}$ and then build a coloring for each conflict-free set *S* as follows:

$$\begin{array}{cc} C(x)=\mathrm{in} & \;\text{if}\;x\in S\\ C(x)=\mathrm{out} & \;\text{if}\;S{↣}^{R}x;\\ C(x)=\mathrm{att} & \;\text{if}\;x{↣}^{R}S\;\text{and }S{↣̸}^{R}x\\ C(x)=\mathrm{ud} & \;\text{otherwise} \end{array}$$

Example 9 Consider the *leaf node* $t_{5}$ with bag {a,b} in Fig. 4(b). The computed colorings represent the conflict-free (and ∅-restricted admissible) sets for $F_{\geq t_{5}}$. For instance, the second labeling *C* with C(a)=att,C(b)=in corresponds to the ∅-restricted admissible/conflict-free set {b}, which however is not admissible for $F_{\geq t_{5}}$.

REMOVAL node: In a removal node we have $X_{t}=X_{t^{\prime }}\setminus \{a\}$ for some node *a*. For each valid coloring of $t^{\prime }$ with C(a)≠att we build a new coloring for node *t* by simply deleting the value for *a* and keeping all the remaining values. As we remove the argument *a*, by the connectedness of tree-decompositions, we know that we have already considered all neighbors of *a*. Now suppose *C* is a valid coloring for $t^{\prime }$, but has C(a)=att, i.e., *a* must be attacked to make the set admissible. As all neighbors of *a* were already considered we know that the corresponding sets cannot be extended to an admissible set and thus we delete this coloring. If C(a)≠att, then *a* does not cause a problem w.r.t. admissibility and as already all neighbors were considered will never do so.

Example 10 Node $t_{4}$ in Fig. 4(b) is a *removal node* with $X_{t_{4}}=X_{t_{5}}\backslash \{a\}$. According to the definition for the computation of colorings in removal nodes the colorings for $t_{4}$ are obtained from the colorings of $t_{5}$ except for the second coloring $C^{\prime }$ (where $C^{\prime }(a)=\mathrm{att}$ and $C^{\prime }(b)=\mathrm{in}$). Here, argument *b* is not defended against the attack from *a*. Therefore, {b} is not an $X_{>t_{4}}$ (or {a})-restricted admissible set for $F_{\geq t_{4}}$.

INTRODUCTION node: For an introduction node we have $X_{t}=X_{t^{\prime }}\cup \{a\}$. We build two colorings C+a and $C\;\overset{˙}{+}\;a$ for *t* as described below. The first is always valid while the second is only valid if ${\left[C\overset{˙}{+}a\right]}_{\mathrm{in}}$ is conflict-free.

$$(C+a)(b)=\left\{ \begin{array}{cc} C(b) & \text{if}\;b\in A\\ \mathrm{out} & \text{if}\;b=a\;\text{and}\;{\left[C\right]}_{\mathrm{in}}{↣}^{R}a\\ \mathrm{att} & \text{if}\;b=a\;\text{and}\;{\left[C\right]}_{\mathrm{in}}{↣̸}^{R}a\;\text{and}\;a{↣}^{R}{\left[C\right]}_{\mathrm{in}}\\ \mathrm{ud} & \text{otherwise} \end{array}\right.$$

$$(C\;\overset{˙}{+}\;a)(b)=\left\{ \begin{array}{cc} \mathrm{in} & \text{if}\;b=a\;\text{or}\;C(b)=\mathrm{in}\\ \mathrm{out} & \text{if}\;a\neq b\;\text{and}\;((a,b)\in F_{t}\;\text{or}\;C(b)=\mathrm{out})\\ \mathrm{ud} & \text{if}\;a\neq b\;\text{and}\;C(b)=\mathrm{ud}\;\text{and}\;(a,b)\notin F_{t}\;\text{and}\;(b,a)\notin F_{t}\\ \mathrm{att} & \text{otherwise} \end{array}\right.$$

In an introduction node we add a new argument to the framework. So for each extension we get two new candidates, one where we leave the argument *a* outside the extension (case C+a) and one where we add *a* to the extension (case $C\;\overset{˙}{+}\;a$). For the first coloring we just have to compute whether to color the new argument by *out*, *att* or *ud* while for the second coloring we first have to check that the set is still conflict-free and if so we have to update the colors of the old arguments according to their attacks with *a*. That is, if $(a,b)\in F_{t}$ then *b* is labeled *out* and if $(b,a)\in F_{t}$ and *b* is not already labeled *out* then it is labeled *att*.

Example 11 In node $t_{3}$ in Fig. 4(b) argument *c* is introduced, i.e. $t_{3}$ is an *introduction node*. Consider the second coloring $C^{\prime }$ of $t_{4}$ where $C^{\prime }(b)=\mathrm{ud}$. Here we have two possibilities for adding *c*. If we do not add *c* to the set of selected arguments we obtain a coloring $C_{1}$ for $t_{3}$ where both arguments *b* and *c* are set to *ud*. On the other hand, by adding *c* to the set of selected arguments we obtain the coloring $C_{2}$ where $C_{2}(b)=\mathrm{out}$ and $C_{2}(c)=\mathrm{in}$. Note that the color of *b* changes in this case from *ud* to *out* as *c* attacks *b*. Furthermore, note that this coloring coincides with the coloring obtained from $C^{\prime \prime }$ of $t_{4}$ with $C^{\prime \prime }(b)=\mathrm{out}$ in case *c* is added to the set of selected arguments. Hence, $C_{2}$ represents both {a,c} and {c} which are $X_{>t_{3}}$ (or {a})-restricted admissible sets for $F_{\geq t_{3}}$.

JOIN node: A JOIN node has two child nodes $t^{\prime },t^{\prime \prime }$ with $X_{t}=X_{t^{\prime }}=X_{t^{\prime \prime }}$. We combine each valid coloring *C* of $t^{\prime }$ with each valid coloring *D* of $t^{\prime \prime }$ such that ${\left[C\right]}_{\mathrm{in}}={\left[D\right]}_{\mathrm{in}}$ and build a new coloring as follows: All arguments in ${\left[C\right]}_{\mathrm{in}}$ are colored *in*. An argument $x\in X_{t}$ is colored with *out* iff one of C,D colors it with *out*. The remaining arguments are colored with *att* iff one of C,D colors it with *att* and *ud* iff both C,D color it with *ud*.

The intuition behind this step is the following. The frameworks $F_{\geq t^{\prime }}$ and $F_{\geq t^{\prime \prime }}$ are different parts of *F* that only intersect on $X_{t}$. So an extension of $F_{\geq t^{\prime }}$ can be combined with an extension of $F_{\geq t^{\prime \prime }}$ as long as they coincide on the intersection. The join rule for the colorings corresponds to the fact that an argument attacks/is attacked by the union of two sets iff it attacks/is attacked by at least one of them.

Example 12 In the *join node* $t_{2}$ in Fig. 4(b) two colorings *C* and *D* are combined in case ${\left[C\right]}_{\mathrm{in}}={\left[D\right]}_{\mathrm{in}}$, i.e., they coincide on their *in*-colored arguments. Consider the second coloring $C^{\prime }$ of $t_{3}$ where $C^{\prime }(b)=\mathrm{out}$ and $C^{\prime }(c)=\mathrm{ud}$ as well as the second coloring $D^{\prime }$ of $t_{6}$ where $D^{\prime }(b)=\mathrm{ud}$ and $D^{\prime }(c)=\mathrm{out}$. Based on the definition of the join operator their combination results in a coloring *C* with C(b)=out and C(c)=out which represents one $X_{>t_{2}}$ (or {a,d,e})-restricted admissible set for $F_{\geq t_{2}}$, namely {a,d}.

#### 4.3.3 Reasoning problems

The dynamic-programming based approach can be used to solve several reasoning problems.

##### Enumerating extensions

In order to enumerate all extensions for a semantics *σ* the tree decomposition is traversed a second time in top-down order after the initial bottom-up computation. Thereby only relevant solutions (the *extensions*) are considered. Note that we do not compute $e_{t}(C)$ explicitly during the first traversal as this would destroy tractability. In particular, it is guaranteed that the second traversal only considers colorings that yield a solution. So enumerating extensions can be done with linear effort for each extension. For our running example AF *F* we obtain ${\mathrm{Enum}}_{\mathrm{adm}}(F)=\{\emptyset ,\{a\},\{d\},\{a,d\}\}$. In Fig. 4(b) this result is represented by the column $e_{t_{0}}(\cdot )$ in node $t_{0}$.

##### Counting extensions

In case we are only interested in the number of extensions a second traversal of the tree decomposition is not necessary. It is sufficient to calculate the number of $X_{>t}$-restricted admissible sets that are represented by the respective coloring immediately during the bottom-up traversal. The columns Cnt in Fig. 4(b) show the number of represented sets for each coloring. Consider for example coloring *C* of $t_{3}$ where C(b)=out and C(c)=in: *C* represents two $X_{>t_{3}}$-restricted admissible sets as it results from the two colorings of $t_{4}$ where each represents one restricted set. At the root node we obtain ${\mathrm{Count}}_{\mathrm{adm}}(F)=4$.

##### Deciding credulous acceptance

Credulous acceptance of an argument *x* can be decided by storing an additional flag together with each coloring: In case C(x) for a coloring *C* is set to *in*, *C* is marked. Additionally, this information is passed upwards the tree: If a coloring is constructed on basis of a marked coloring it is marked as well. Finally, in case the coloring at the root node is marked, we know that *x* is credulously accepted. In Fig. 4(b) this is represented by the columns Crd where we want to decide whether *a* is credulously accepted. For ${\mathrm{Cred}}_{\mathrm{adm}}(a,F)$ we obtain *yes*. For skeptical acceptance, a dual approach can be employed (see [40]).

#### 4.3.4 Problems beyond NP

So far we have only considered admissible semantics but the dynamic programming approach is in no way limited to problems that are in NP. Harder problems, however, generally need a more complicated data structure. Consider preferred semantics where, for example, deciding ${\mathrm{Skept}}_{\mathrm{prf}}$ is known to be $\Pi_{2}^{P}$-complete. We only give a rough outline of the ideas to extend the above algorithm for preferred semantics, for details the interested reader is referred to [40].

As preferred extensions are subset-maximal admissible sets in order to guarantee subset maximality one can use pairs (C,Γ) as a data structure within a node *t* instead of colorings. Here, *C* is a coloring and *Γ* is a set of colorings, called *certificates*. The certificates characterize all $X_{>t}$-admissible sets which are strictly larger than the $X_{>t}$-admissible sets characterized by *C*. One can consider *Γ* as counter-examples for *C* representing subset-maximal $X_{>t}$-admissible sets. During the traversal of the tree decomposition, the colorings and certificates are computed analogously to the colorings for admissible semantics. At the root node *r*, one checks for each pair (C,Γ) whether Γ=∅. If this is the case, *C* represents subset-maximal $X_{>r}$-admissible sets, which correspond to preferred extensions.

## 5 System comparison

In this section we provide an overview on systems that implement the approaches presented earlier. We focus here on a comparison of the systems w.r.t. their features (e.g. supported semantics and reasoning problems) and underlying concepts. Our goal is to provide a comprehensive study of the strengths of each tool, where the reader can look up the appropriate tool for the problem at hand. The features of the presented systems are naturally subject to change in the future. We note that the landscape of currently available software is very heterogeneous: Some tools are tailored to graphical representations of the used algorithms and results whereas others are particularly tuned towards performance. Within the argumentation community there is currently no consensus on which instances are representative for comparing different implementations w.r.t. performance. Moreover, independent benchmark suites are not available. Hence, a systematic, fair and longer-term stable comparison w.r.t. run-time performance is currently not possible, and we refer to currently ongoing developments within the community that seek for a standardized system competition of argumentation systems [48,123,43]. Nevertheless, where available, we give references to articles that deal with a performance comparison of particular tools.

Table 2 summarizes systems for abstract argumentation. The URL links to the web page of the respective system, where source code and documentation or the web front-end (if available) can be found. Additionally, the table contains a reference to the section where the algorithms underlying the tool are discussed. The last column contains the reference to the main article of the tool. In case no particular article on the tool was published, we reference here the paper that presents the theoretical background of the tool.

Table 3 lists the technical characteristics of the considered systems. The “GUI” column not only indicates the availability of a full-fledged graphical user interface but also contains information about availability of front-ends for demonstration purposes. Column “command line” denotes that the software is accessible via command line interface, and “library” specifies that the implementation can be accessed via a specified software interface.

In Table 4 we provide an overview on the supported semantics and reasoning problems of the systems. Note that this table only contains the semantics and reasoning problems we consider throughout this work (see Section 2). Additionally, we also include *implicit* reasoning support in the table, denoted by the italic letters *C* and *S*. That is, we know that credulous reasoning yields the same answer for any AF and argument w.r.t. preferred, complete and admissible semantics. Similarly, skeptical reasoning for complete and grounded semantics return the same result. Since the grounded extension is unique, credulous and skeptical reasoning are equivalent for grounded semantics.

The strengths of each tool are summarized in Section 5.1. There, we go into detail of system-specific characteristics, such as particular GUI-based features, support for additional reasoning problems or performance-relevant details. If a system is capable of computing further semantics, such as ideal [124], eager [125], cf2 [126], stage2 [127], resolution-based grounded [128] we note this in the corresponding system paragraph.

### 5.1 System properties

##### ArgSemSAT

The high-performance system ArgSemSAT is built on top of modern SAT solvers in such that it incorporates an iterative SAT-procedure. In particular, it implements the PrefSat approach [82] from Section 3.1.3. The procedure relies on iteratively generating complete extensions/labelings and extending them iteratively to preferred extensions. A similar approach is taken by CEGARTIX, where skeptical acceptance of preferred semantics (among other query-based reasoning problems) is computed. The implementation of PrefSat showed good performance compared to ASPARTIX (even with its metasp approach) and ArgTools [82]. ArgSemSAT allows to choose between different SAT-solvers and processes input in the ASPARTIX input format. In future it is also supposed to include direct algorithms based on the SCC-recursive schema [22,129].

##### ArgTools

This system aims to provide a fast implementation of a labeling-based algorithm for enumerating all preferred extensions (cf. Algorithm 4). While the main focus of the research behind this tool is directed towards efficient enumeration of preferred semantics [36] there are several other results. First, enumeration algorithms for several other semantics, i.e. those depicted in Table 4 and ideal, where developed in [105]. Second, in [36] the authors present an implementation of optimizations for credulous and skeptical reasoning with preferred semantics. This line of research compares the performance of different labeling based algorithms and in particular gives empirical evidence, by comparing the algorithms on randomly generated instances, that the newly proposed algorithms are the fastest ones. Some of the algorithms are also compared with ASPARTIX (using the DLV solver) and dynPARTIX where ArgTools again showed good performance.

##### ASPARTIX

The “Answer Set Programming Argumentation Reasoning Tool” [30] is based on reductions to ASP as discussed in Section 3.3. It consists of a collection of ASP encodings, where each encoding, augmented by a given AF in form of ASP facts, can be given as input to an ASP solver in order to compute the extensions. For most semantics, ASPARTIX provides encodings for the solver DLV as well as gringo/clasp(D). Following the reduction approach, ASPARTIX's performance scales with new versions of these solvers. Furthermore, the system is platform independent in the sense that it runs on any system supporting the ASP solvers. ASPARTIX also offers a web front-end, where any argumentation framework as well as its extensions can be inspected graphically. A particularly useful feature of this system is that it supports many semantics and solves various reasoning problems. In addition to the semantics in Table 4, ASPARTIX supports ideal, cf2, stage2 and resolution-based grounded semantics. Since ASP solvers support enumeration and credulous as well as skeptical query-based reasoning, this can directly be utilized by ASPARTIX. The system is often used as a reference system in performance comparisons [21,82,31,36,37,83,98,105,130,45,44,131].

##### CEGARTIX

The “Counter-Example Guided Argumentation Reasoning Tool” [21] is built on top of modern SAT solvers, and relies on an iterative procedure of SAT-calls (see Algorithm 1). As a command-line tool, CEGARTIX is built towards performance and computes the skeptical acceptance of an argument w.r.t. preferred, semi-stable and stage semantics and for the last two also credulous acceptance. Like ArgSemSAT [82], which relies on iterative SAT calls for enumerating preferred labelings, CEGARTIX can be seen as a sort of hybrid approach between direct and reduction-based methods, since only certain sub-tasks are delegated to a SAT solver. CEGARTIX is available online as a binary. The system allows the user to configure which SAT-solver she wants to use. Being based on a reduction approach, CEGARTIX scales with newer versions of SAT solvers and the system has been shown to be competitive w.r.t. ASPARTIX [21] and also processes the ASPARTIX input format.

##### CompArg

CompArg [38] is intended for determining credulous acceptance of arguments w.r.t. preferred semantics and enumerating grounded, preferred, stable and semi-stable extensions. It implements the labeling-based approach as presented in Section 4.1 (Algorithm 4). Written in Delphi, the executable for Windows is publicly available. The system comes with many examples, which suits its main educational aim of illustration of the semantics. Due to this purpose it is not primarily built for high performance. The tool consists of a GUI that illustrates the computation of the acceptance status, either by providing proofs or refutations of arguments. Additionally, several example instances are provided. Therefore, CompArg is particularly useful when it comes to get a deeper understanding of the underlying algorithm. Besides deciding credulous acceptance, the resulting extensions can be enumerated.

##### ConArg

The system ConArg [26] follows a reduction-based approach towards CSPs as presented in Section 3.2. Internally, the tool uses sophisticated Java implementations of CSP engines (JaCoP). Its performance thus scales with newer versions of these engines, and it is platform-independent through the use of Java. It supports the enumeration of extensions for many semantics (see Table 4) and is capable of verifying whether a given set is a preferred extension. The tool features a simple and intuitive graphical user interface for inspecting the AF and the extensions at hand. It supports the ASPARTIX input format for AFs, and also allows to generate random (weighted) argumentation frameworks. ConArg is also available as a web-interface with an interactive graphical representation. Recently, the second version of ConArg with some modifications was released [131,43,44]. To improve the performance, ConArg2 is now based on Gecode, an efficient C++ environment for constraint-based applications. ConArg2 is available as a pre-compiled command line tool for Linux. Besides the features of ConArg, ConArg2 also allows for credulous and skeptical reasoning for admissible, stable and complete semantics. ConArg and ConArg2 showed good performance compared to ASPARTIX and Dung-O-Matic [130,131].

##### Dung-O-Matic

Dung-O-Matic is based on dialectical proof-procedures (see Section 4.2) and includes implementations for many different semantics. Besides support for most of the semantics listed in Table 4, ideal and eager extensions for a given AF can be computed. Implemented as a Java library, it can be flexibly used across platforms. For demonstration purposes, it is accessible via the tool OVAgen.^15^ OVAgen is a web-based software where argumentation frameworks can be drawn graphically and the resulting extensions are visualized. A preliminary performance comparison against ASPARTIX and ConArg is published by Bistarelli et al. [130]. Although this work shows that the tool is outperformed by the other two systems when computing complete or stable extensions, one has to note that further comparisons and, in particular, real-world instances are necessary to gain a better picture of the tool's performance.

##### Dungine (part of ArgKit)

Dungine [109] implements algorithms based on dialogue games, and currently provides native support for grounded and preferred semantics. It is part of ArgKit, a Java library intended for building custom software based on argumentation. For demonstration purposes, the ArgKit package includes examples of GUI applications. Additionally, similar to Dung-O-Matic, the software is integrated in the tool OVAgen. Since the source code is made publicly available under the LGPL license, it can be integrated in other projects.

##### dynPARTIX

The concept underlying the “Dynamic Programming Argumentation Reasoning Tool” [42,115] is based on dynamic programming, where the instance is decomposed before solving (see Section 4.3). The tool exploits the structure of the given argumentation framework, where the decomposition is constructed based on heuristics. Hence, its run-time performance is particularly good for instances with tree-like structures. The tool, implemented in C++, is currently available as Linux executable. A special characteristic that differentiates it from the other systems presented here is its ability to provide the overall number of solutions without explicit enumeration.

##### PyAAL (+ArguLab)

The “Python Abstract Argumentation Library” implements labeling-based procedures for determining the justification status of arguments and for enumerating the labelings for many semantics (see Section 4.1). In addition to the functionality summarized in Table 4, PyAAL is able to compute the ideal and eager labeling, as well as determining the corresponding justification status. ArguLab [107] is a web front-end that allows to demonstrate the capabilities of PyAAL. Within ArguLab, in a first step the argumentation framework is constructed. Next, based on the selected semantics the labelings associated with the arguments are visualized. The tool allows to interactively analyze the justification status of arguments. Note that ArguLab is designed for demonstration purposes only, but the underlying code of PyAAL can be used without restrictions (GPL licensed). Its particular strength lies in the fact that it supports a broad number of semantics and solves several reasoning problems.

### 5.2 Summary

The system comparison illustrates the diverse landscape of available tools for abstract argumentation: While some systems cover a wide range of different semantics (e.g., ASPARTIX, ConArg, Dung-O-Matic, and PyAAL), others are well-suited for illustration and demonstration purposes of the algorithms (e.g., CompArg and Dungine) or are tailored towards solving particular problems efficiently (e.g., ArgSemSAT, ArgTools, CEGARTIX and dynPARTIX). Also, diversity is observable when considering the supported semantics and solvable reasoning problems (see Table 4). Among the considered systems, no semantics and reasoning problem is supported by all tools. Additionally, to promote their functionality, several tools provide access to their systems via a web interface (e.g., ASPARTIX, ConArg, Dung-O-Matic, Dungine and PyAAL), which allows to test the system without the necessity to download or install software.

The available run-time comparisons do not indicate that one system outperforms all others. However, we observed that ASPARTIX is, in most cases, used as a base line system for performance comparisons. To give a clearer picture on the performance aspects of the tools, there is a need for independently created and publicly available benchmark suites. This (and also ideas on running even a public system competition for argumentation systems) is discussed within the community (see, e.g., [132]).

Additionally, besides run-time performance, many other aspects are important for a good system, including intuitive design, versatility, extendability, and also source code availability or ongoing support and development of the system. Each tool has its unique characteristics and advantages, therefore the choice for the right tool mainly depends on the problem at hand.

## 6 Discussion

We conclude our survey on implementation of abstract argumentation with various issues we have not touched yet. This includes methods for further semantics (Section 6.1) and complementary aspects for evaluating abstract argumentation frameworks, for instance, pre-processing (Section 6.2). In Section 6.3, we give pointers to systems which are in a certain way concerned with abstract argumentation, but have a more general aim (in fact, methods as presented in this survey could be used *within* such systems). We then proceed with a global summary and discuss directions which we believe are important for future developments.

### 6.1 Further semantics

In the interest of space, we have omitted a few prominent semantics in the main body of this survey. In what follows we give respective pointers to the literature and highlight systems implementing these semantics.

As shown by Baroni et al. [126] argumentation semantics can be defined on the basis of decomposing an AF into its strongly connected components (SCCs). This not only provides alternative definitions of some of the semantics which we have already discussed in the paper, but also leads to novel semantics, for instance cf2 [126] and stage2 [127] semantics. For both semantics, ASP encodings [127,133] as well as labeling-based algorithms [127] have been presented, the former are integrated in the ASPARTIX system.

Moreover, there is the family of resolution-based semantics [128], with the resolution-based grounded semantics being the most popular instance. Different ASP encodings for resolution-based grounded semantics are studied in [31] and are incorporated in the ASPARTIX system, as well.

Finally, the unique-status semantics ideal [124] and eager [125] (for a general notion of parametric ideal semantics, see [134]) have been proposed to perform a prudent form of reasoning on the set of preferred extensions and semi-stable extensions, respectively. A characterization in terms of labelings for ideal and eager semantics is given in [135] and labeling-based algorithms have been implemented in the ArguLab system. Also the Dung-O-Matic system allows for reasoning with ideal and eager semantics. In the ASP-setting a characterization for ideal semantics is given in [30] and is implemented in the ASPARTIX system. Regarding other reduction-based systems, ConArg is also capable of computing the ideal extension of an AF.

### 6.2 Further methods

Next, we briefly describe three concepts which can be considered to be used on top of argumentation systems as discussed in this survey. These methods can be seen as pre-processing or simplification steps before actually evaluating abstract argumentation frameworks.

First, the idea of splitting allows to divide an argumentation framework *F* in (two) smaller argumentation frameworks $F_{1}$, $F_{2}$, such that there are no attacks from arguments in $F_{2}$ to arguments in $F_{1}$ [136,137]. Then one can first compute the extensions of $F_{1}$ and then for each of its extension *E* compute the extensions for a slightly modified version $F_{2}^{E}$ of $F_{2}$. The extensions of *F* can then be obtained by combining each extension *E* of $F_{1}$ with the extensions of the frameworks $F_{2}^{E}$. The benefit from this splitting approach comes from the fact that both $F_{1}$ and $F_{2}$ are smaller than the original AF *F* and thus can be evaluated faster (however, in the worst case an exponential number of AFs $F_{2}^{E}$ has to be handled). The idea of splitting AFs has also been generalized by allowing a small number of attacks from arguments in $F_{2}$ to arguments in $F_{1}$, see [138]. In a recent paper, Liao and Huang have proposed a related method to evaluate only parts of a given framework when it comes to credulous or skeptical reasoning problems [139].

Second, the identification of redundant patterns might be used to simplify argumentation frameworks before evaluation. The notion of strong equivalence [73,140] provides means to identify redundant attacks without analyzing the entire framework (an example are attacks between two self-attacking arguments; such attacks can be safely removed for most of the semantics). Relaxed notions of strong equivalence might be even more beneficial for this purpose, see, e.g., [141,142].

Finally, we mention the concept of intertranslatability between abstract argumentation semantics [72]. Here, one is interested in translations from a semantics *σ* to another semantics *τ*, i.e., a function *Tr* that transforms arbitrary argumentation frameworks *F* such that σ(F)=τ(Tr(F)). If this translation function *Tr* can be computed efficiently we can combine it with any system for semantics *τ* to build a system for *σ*. So translations between different semantics allow to expand the applicability of existing argumentation systems.

### 6.3 Further systems

In this work we focused on systems that implement the evaluation of semantics on Dung's abstract argumentation framework directly. However, there exists a wide range of systems that extend these capabilities, in particular by additionally supporting instantiation of argumentation frameworks.

One approach is based on ASPIC [143], resp. ${\mathrm{ASPIC}}^{+}$ [50], which instantiates Dung-style frameworks. Arguments are represented as inference trees by applying strict and defeasible inference rules. TOAST (The Online Argument Structures Tool) [144] is an implementation of ${\mathrm{ASPIC}}^{+}$ and is available as web front-end.^16^ The user-specified knowledge base, rule set, contrariness and preferences are used to construct an argumentation system which can currently be evaluated based on grounded, preferred, semi-stable and stable semantics. The ASPIC argumentation engine demo^17^ implements several instantiations of ASPIC and provides a web interface. Again the user can specify a knowledge base and a rule set to construct an argumentation system which then can be evaluated based on grounded and credulous preferred semantics. The Carneades Web Service^18^ is capable of “argument construction, storage, navigation, querying, evaluation, visualization and interchange” [145]. It is based on the ${\mathrm{ASPIC}}^{+}$ model of structured argument but still preserves the features of the original version of Carneades system [51]. On the resulting Dung-style framework it applies grounded semantics. An approach based on classical logic and argument instantiation is shown in [146]. Here, arguments and possible counterarguments are constructed from a classical propositional knowledge base. Furthermore, Vispartix^19^ consists of a collection of ASP encodings [147] for obtaining Dung argumentation frameworks from a propositional knowledge base (and a set of predefined claims), based on the approach presented in [148]. The argumentation framework can then, for example, be evaluated by ASPARTIX. Another survey [54] summarizes systems that focus on the construction of arguments. This includes approaches based on classical [148] and defeasible logic [53] and briefly introduces the systems ASPIC and CaSAPI^20^ (which combines abstract and assumption-based argumentation).

There is also recent work on translating different argumentation models. In [149], a translation between Carneades and Dung AFs is studied and implemented in the functional programming language Haskell. The benefit of such systems is that one may re-use engines for AFs to compute results for Carneades and potentially other argumentation models. Regarding different argumentation models, a reduction-based approach was implemented for recursive, probabilistic defeasible logic programming (RP-DeLP). The resulting system uses ASP for computing the results [150].

Finally, a recent review on systems for argumentation in the Social Semantic Web [151] summarizes social web tools, and discusses how argumentation can be modeled in this context. It contains an exhaustive study of online tools that combine Web 2.0 and Semantic Web technologies. In the course of the review, it gives an comparative overview on current developments of practically applied argumentation research on the web.

### 6.4 Summary

The aim of this article was to provide the reader with a basic understanding of the different techniques used to implement the paradigm of abstract argumentation. We have grouped these techniques into two categories. The reduction-based techniques aim at transforming the argumentation problem at hand into an instance of a different problem (SAT, ASP, etc.), thereby delegating the burden of computation to existing systems. On the other hand, the category of direct approaches refers to systems and methods implementing abstract argumentation “from scratch”, thus allowing for tailored algorithms which explicitly realize argumentation specific optimizations. The strengths of reduction-based systems are that they (i) directly scale with newer versions of solvers and (ii) can be easily adapted to specific needs, which is mirrored by a quite flexible support of reasoning problems and argumentation semantics. Ultimately, dedicated direct algorithms (when tuned sufficiently) outperform reduction-based ones (see e.g. [36]), but each reasoning problem and semantics needs its own full-fledged implementation. In conclusion, this suggests that the reduction-based approach provides a good basis for rapid prototyping, while systems that have to scale well for large problems require substantial design efforts.

However, the two categories are not as strictly separated as it might look like. For instance, the CEGARTIX approach as introduced in Section 3.1.3 is an example for a system that combines the advantages of the two categories. It is based on a dedicated algorithm for the argumentation problem at hand, but as a subroutine invokes existing systems (i.e., SAT solvers). Systems that combine these approaches are among the fastest systems, in particular significantly faster than pure reduction-based approaches and still directly benefit from improvements in the solvers. However, by this hybrid approach one loses the flexibility of pure reduction-based systems.

The current landscape of systems is very heterogeneous (see Section 5). Available systems for argumentation differ in their support for semantics and reasoning problems, summarized in Table 4. Each tool has its unique strengths and characteristics, be it graphical representation of algorithms or results, efficient solving in some setting, or providing broad support for problems to be solved in the context of argumentation.

### 6.5 Future directions

Although significant progress has been made in the last years in implementing efficient systems for abstract argumentation, there is still a wide range of open issues.

On the one hand, several optimization methods which proved successful in other areas still have to be adapted for abstract argumentation systems. Methods including symmetry breaking, parallelization, heuristics and algorithm selection come to our mind. Likewise, the area of average-case and probabilistic algorithms has not been considered in combination with abstract argumentation yet. Even more important, benchmark suites are needed to evaluate and witness the value of such optimizations and, more generally, to compare the different approaches which are nowadays available on a broad and objective scope. To date, experiments are typically performed on some randomly generated frameworks. However, a better theoretical model for such frameworks is required in order to have a more meaningful picture when runtimes are measured. On the other hand, collections of structured instances stemming from real-world applications domains are lacking. Several ideas for establishing a benchmark library for abstract argumentation have first been collected in [152] and the upcoming first competition for argumentation systems, http://argumentationcompetition.org, will be very beneficial for the evolution of systems.

Moreover, we have to understand particularities in the argumentation domain to tune the systems towards more practical needs, in particular when used within an instantiation-based argumentation context. First, argumentation is inherently dynamic [153–155] and thus one expects that argumentation frameworks are continuously evolving. Consequently, methods which allow for incremental evaluation of frameworks (i.e., the system “remembers” the framework it has evaluated last time and tries to build the current solving on this prior results) are an important research direction. A first valuable theoretical contribution in this direction can be found in [156]. Second, many people in the community argue that abstract argumentation is not a stand-alone formalism. Consequently, the integration of “abstract” into “real” argumentation systems is central. In particular, the specific needs of these real argumentation systems have to be taken into account when abstract argumentation systems are improved. To this end, it has to be clarified whether such integrated systems lead to abstract frameworks of certain structure (in particular, in many cases, instantiations lead to particular symmetries in the resulting frameworks). Advanced abstract argumentation systems therefore should either be optimized towards such structures or provide interfaces which allow to feed additional information from the instantiation process to the system in order to guide heuristics or to prune the search space.

In conclusion, we believe that the challenge of implementing abstract argumentation systems is a perfect play-ground to apply and test different techniques on a set of uniform yet computationally manifold problems which are given by the different semantics for abstract argumentation. The future will show which techniques prove successful or whether completely novel methods will emerge in course of these investigations.

## Figures and Tables

**Algorithm 1:**
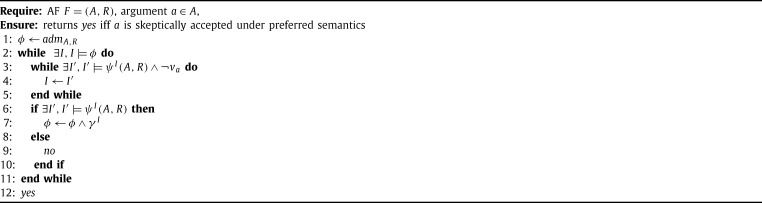
${\mathrm{Skept}}_{\mathrm{prf}}(a,F)$.

**Algorithm 2:**
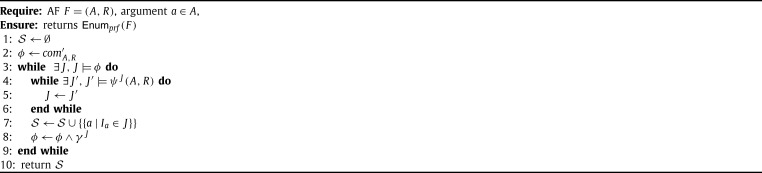
${\mathrm{Enum}}_{\mathrm{prf}}(F)$.

**Algorithm 3:**
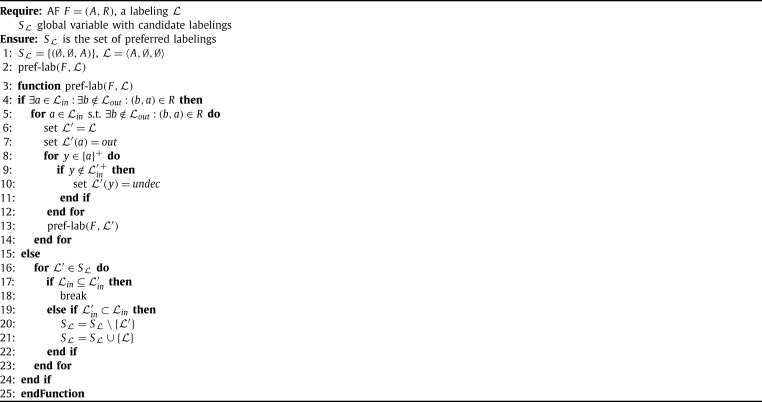
pref-lab(F).

**Algorithm 4:**
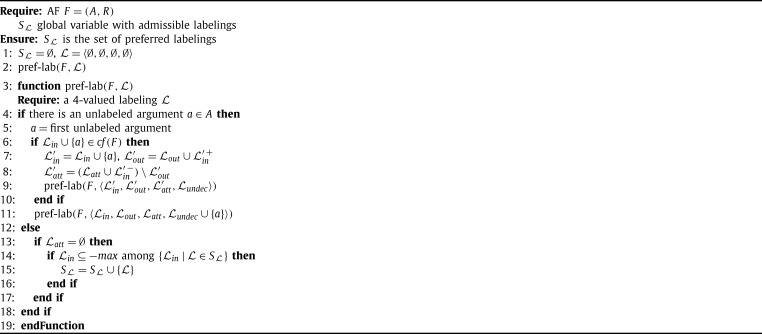
pref-lab(F).

**Algorithm 5:**
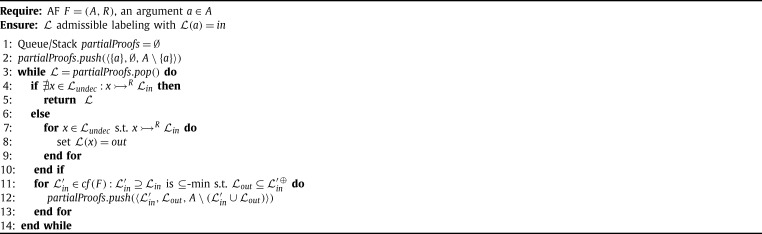
cred-pref(F,a).

**Table 1:** Computational complexity of reasoning in AFs.

*σ*	${\mathrm{Cred}}_{\sigma }$	${\mathrm{Skept}}_{\sigma }$	${\mathrm{Ver}}_{\sigma }$
*naive*	in L	in L	in L
*stb*	NP-c	coNP-c	in L
*adm*	NP-c	trivial	in L
*com*	NP-c	P-c	in L
*grd*	P-c	P-c	P-c
*prf*	NP-c	$\Pi_{2}^{P}$-c	coNP-c
*sem*	$\Sigma_{2}^{P}$-c	$\Pi_{2}^{P}$-c	coNP-c
*stg*	$\Sigma_{2}^{P}$-c	$\Pi_{2}^{P}$-c	coNP-c

**Table 2:** Argumentation systems: overview.

System name	URL	Section (type)	Reference
ArgSemSAT	http://sourceforge.net/projects/argsemsat/	3.1.3 (reduction, iterative SAT)	[82,22]
ArgTools	http://sourceforge.net/projects/argtools/	4.1 (direct, labelings)	[36]
ASPARTIX	http://www.dbai.tuwien.ac.at/proj/argumentation/systempage/	3.3 (reduction, ASP)	[30]
CEGARTIX	http://www.dbai.tuwien.ac.at/proj/argumentation/cegartix/	3.1.3 (reduction, iterative SAT)	[21]
CompArg	http://www.ai.rug.nl/~verheij/comparg/	4.1 (direct, labelings)	[38]
ConArg	http://www.dmi.unipg.it/conarg/	3.2 (reduction, CSP)	[26]
Dung-O-Matic	http://www.arg.dundee.ac.uk/?page_id=279	4.2 (direct, dialogue)	–
Dungine (ArgKit)	http://www.argkit.org/	4.2 (direct, dialogue)	[109]
dynPARTIX	http://www.dbai.tuwien.ac.at/proj/argumentation/dynpartix/	4.3 (direct, decomposition)	[115]
pyAAL (+ArguLab)	http://code.google.com/p/pyafl/	4.1 (direct, labelings)	[107]

**Table 3:** Argumentation systems: technical details.

System name	Platform	Language	GUI	Command line	Library
ArgSemSAT	independent	C++	–	yes	no
ArgTools	independent	C++	–	yes	no
ASPARTIX	as ASP solver	ASP	web	yes	no
CEGARTIX	Unix	C++	–	yes	no
CompArg	Windows	Delphi	stand-alone	no	no
ConArg	independent	Java, C++	stand-alone	yes	yes
Dung-O-Matic	independent	Java	web (via OVAGen)	no	yes
Dungine (ArgKit)	independent	Java	web (via OVAGen), demo GUI	no	yes
dynPARTIX	Unix	C++	–	yes	no
PyAAL (+ArguLab)	independent	Python	web (ArguLab)	yes	no

**Table 4:**
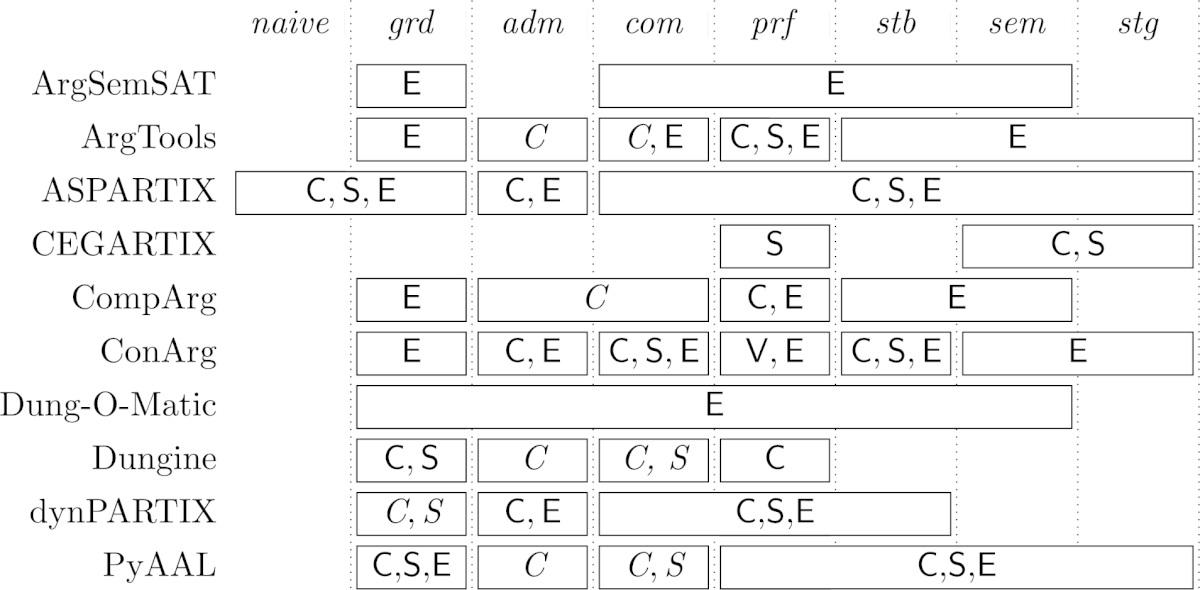
System capabilities showing which system can reason C(redulously), S(keptically) or is able to E(numerate), respectively V(erify) a solution. Implicit reasoning support is denoted by the italic letters *C* and *S*.

## Footnotes

1 http://www.comma-conf.org/.

2 http://homepages.abdn.ac.uk/n.oren/pages/TAFA-13/.

3 http://www.tandfonline.com/toc/tarc20/current.

4 During the presentation of [11] at COMMA 2006.

5 However, in connection with particular instantiation schemes, it is often claimed that only semantics that follow the principle of admissibility (arguments shall only be jointly accepted if each of the selected arguments is defended by the selected set; we will make the concepts more clear in Section 2) should be considered (see, e.g., [5]).

6 See http://argumentationcompetition.org for further information.

7 An overview on these approaches is given in [54].

8 A brief overview on such approaches is given in [55].

9 A similar approach was recently realized for abstract dialectical frameworks (and thus also for AFs) in the system QADF: http://www.dbai.tuwien.ac.at/proj/adf/qadf/.

10 For this technique one should use gringo version 3.0.5 or lower, as later versions don't support the - -reify option.

11 http://www.fico.com/en/products/fico-xpress-optimization-suite/.

12 In order to present several algorithms in this survey in a uniform and easily comparable way, we use different names for the labels compared to the original works [34,36].

13 http://www.wietskevisser.nl/research/epr/.

14 Notice that we use different names for the colors than the original work [112]. This is to present the dynamic programming algorithm in a uniform setting with the labeling-based algorithms presented before.

15 http://ova.computing.dundee.ac.uk/ova-gen/.

16 http://www.arg.dundee.ac.uk/toast/.

17 http://aspic.cossac.org/ArgumentationSystem/.

18 http://carneades.github.com/.

19 http://www.dbai.tuwien.ac.at/proj/argumentation/vispartix/.

20 http://www.doc.ic.ac.uk/~ft/CaSAPI/.
